# Hepatocyte integrity depends on c-Jun-controlled proliferation in *Schistosoma mansoni* infected mice

**DOI:** 10.1038/s41598-023-47646-z

**Published:** 2023-11-21

**Authors:** Lukas Härle, Verena von Bülow, Lukas Knedla, Frederik Stettler, Heike Müller, Daniel Zahner, Simone Haeberlein, Anita Windhorst, Annette Tschuschner, Monika Burg-Roderfeld, Kernt Köhler, Christoph G. Grevelding, Elke Roeb, Martin Roderfeld

**Affiliations:** 1 Department of Gastroenterology, Justus Liebig University Giessen, Gaffkystr. 11c, 35392 Giessen, Germany; 2 Central Laboratory Animal Facility, Justus Liebig University Giessen, 35392 Giessen, Germany; 3 Institute of Parasitology, BFS, Justus Liebig University Giessen, 35392 Giessen, Germany; 4 Institute of Medical Informatics, Justus Liebig University Giessen, 35392 Giessen, Germany; 5 Hochschulen Fresenius GmbH, University of Applied Sciences, 65510 Idstein, Germany; 6 Institute of Veterinary Pathology, Justus Liebig University Giessen, Giessen, Germany

**Keywords:** Diseases, Gastrointestinal diseases, Liver diseases, Parasitic liver diseases

## Abstract

Schistosomiasis is a parasitic disease affecting more than 250 million people worldwide*.* The transcription factor c-Jun, which is induced in *S.* *mansoni* infection-associated liver disease, can promote hepatocyte survival but can also trigger hepatocellular carcinogenesis. We aimed to analyze the hepatic role of c-Jun following *S.* *mansoni* infection. We adopted a hepatocyte-specific c-Jun knockout mouse model (Alb-Cre/c-Jun loxP) and analyzed liver tissue and serum samples by quantitative real-time PCR array, western blotting, immunohistochemistry, hydroxyproline quantification, and functional analyses. Hepatocyte-specific c-Jun knockout (c-Jun^Δli^) was confirmed by immunohistochemistry and western blotting. Infection with *S.* *mansoni* induced elevated aminotransferase-serum levels in c-Jun^Δli^ mice. Of note, hepatic *Cyclin D1* expression was induced in infected c-Jun^f/f^ control mice but to a lower extent in c-Jun^Δli^ mice. *S.* *mansoni* soluble egg antigen-induced proliferation in a human hepatoma cell line was diminished by inhibition of c-Jun signaling. Markers for apoptosis, oxidative stress, ER stress, inflammation, autophagy, DNA-damage, and fibrosis were not altered in *S.* *mansoni* infected c-Jun^Δli^ mice compared to infected c-Jun^f/f^ controls. Enhanced liver damage in c-Jun^Δli^ mice suggested a protective role of c-Jun. A reduced Cyclin D1 expression and reduced hepatic regeneration could be the reason. In addition, it seems likely that the trends in pathological changes in c-Jun^Δli^ mice cumulatively led to a loss of the protective potential being responsible for the increased hepatocyte damage and loss of regenerative ability.

## Introduction

As one of the most important parasitic infections worldwide, schistosomiasis has remained a public health problem for decades. In 2021, more than 250 million people needed preventive treatment in 78 countries, as stated by the World Health Organization (WHO)^1^. Schistosomiasis is depicted as a neglected tropical disease (NTD) mostly common in poor areas in the Middle East, South America, Southeast Asia, and in sub-Saharan Africa^2^. Through climate change and globalization, schistosome parasites expand their original tropical habitats and invade areas with rather moderate climates such as Corsica (France)^3,4^ or Almeria (Spain)^5^.

The cercariae, the infectious larval stage of schistosomes, reside in freshwater and penetrate the human skin upon contact. Among other vertebrate final hosts, humans are a main host, in which cercariae reach the blood vessel system following penetration to develop via a juvenile stage, the schistosomulum, to the adult stage^2^. The genus *Schistosoma* divides into different species such as *Schistosoma mansoni* and *S. japonicum*, both substantially affecting the bowel and liver, as well as *S. haematobium*, which essentially damages the bladder. Our study focused on the species *S. mansoni*^6^. Schistosomes are the only trematodes that have evolved separate sexes. Male and female schistosomes couple and migrate into the mesenteric veins to produce eggs. These eggs can either transmit to the gut lumen to be excreted to the environment, or they get swept away by the bloodstream. Thereby, the eggs reach different organs such as gut, spleen, and liver where they get trapped^7^. Inside these organs, *S. mansoni* eggs cause granuloma formation as a result of the host´s immune reaction^8^. *S.* *mansoni*-induced hepatic granuloma formation is associated with a fibrotic remodeling of the tissue and eventually liver cirrhosis with portal hypertension, splenomegaly, and collateral venous circulation^9^.

The International Agency for Research on Cancer (IARC) categorized *S. mansoni* as a group 3 carcinogen, which indicates an unclear carcinogen status and further research needed^10^. Former studies suggested that *S. mansoni* elevates the pathology and also the risk of developing hepatocellular carcinoma (HCC) in combination with other liver diseases such as chronic Hepatitis B or Hepatitis C^11,12^.

c-Jun is a transcription factor (TF) and a protooncogene in HCC formation^12,13.^ As a part of the activator protein-1 (AP-1) complex, c-Jun can either dimerize as homodimers, or with other members of the AP-1 family as heterodimers. In both cases, c-Jun and its partners bind to DNA with a basic leucine-zipper domain and regulate gene transcription^14^.

The liver of mice lacking c-Jun exhibited impaired proliferation and survival of postnatal hepatocytes, which indicated an important role of this TF in this context^15^. Following partial hepatectomy, the regeneration of mouse livers was also hindered by impaired cell-cycle progression in the absence of c-Jun^16^. This TF was also able to promote hepatocyte survival under different cell-stress scenarios such as ER-stress linked with autophagy^17^ and oxidative stress in acute hepatitis^18^. In our previous work, we demonstrated that S. mansoni eggs^19^ activate the transcription factor c-Jun in hepatocytes by egg-secreted factors such as IPSE, one well-characterized compound of the soluble egg antigens (SEA)^20^. The aim of this study was to examine the function of c-Jun in hepatocytes of *S.* *mansoni-*infected mice.

## Results

### Validation of c-Jun knockout

**Figure 1:**
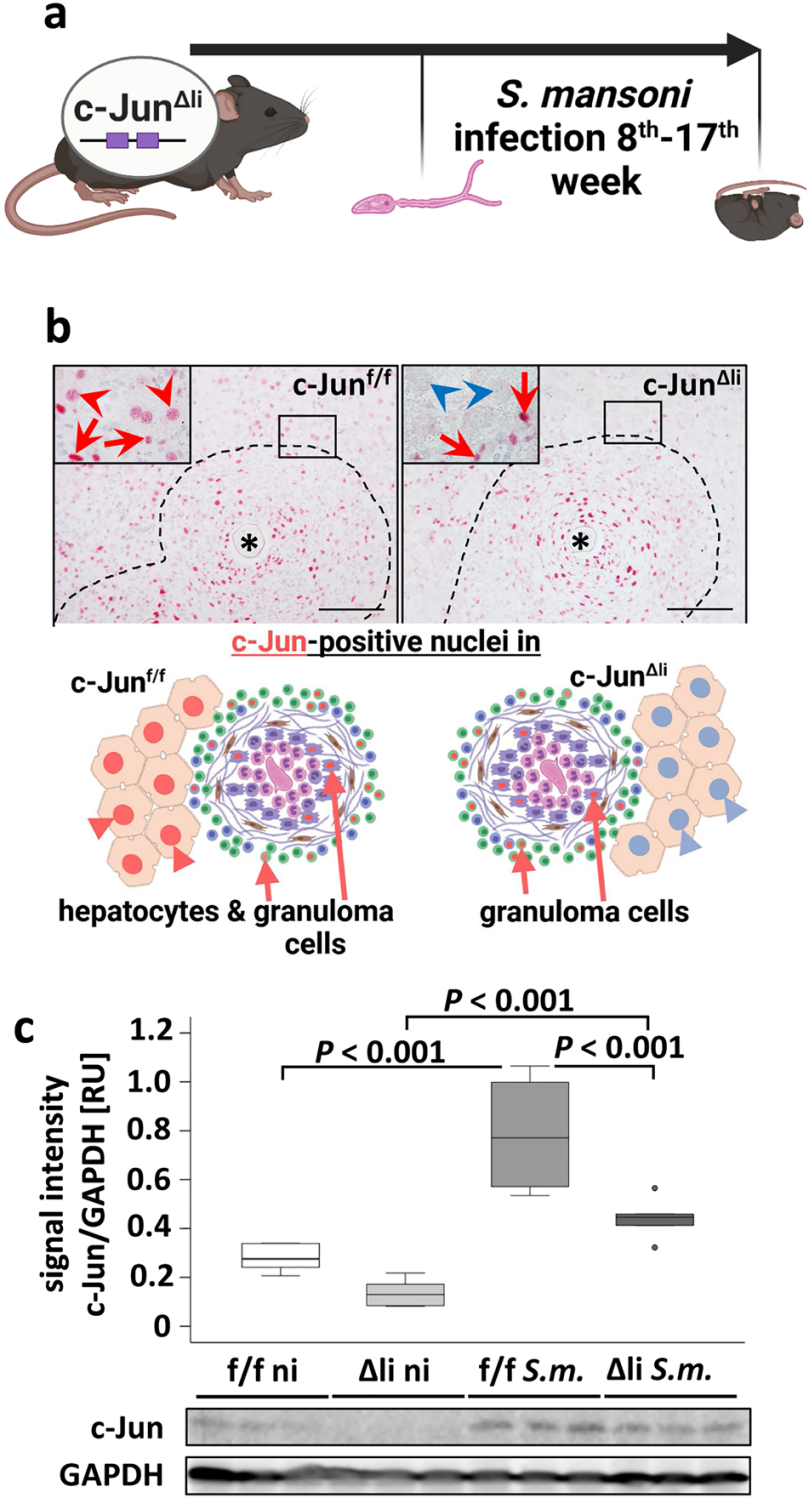
Functional characterization of c-Jun signaling in c-Jun^Δli^ mice infected with the parasite *S.* *mansoni*. (**A**) Schematic illustration of the animal experiment. (**B**) Immunohistochemical staining of c-Jun demonstrated a successful hepatocyte-specific knockout. Red arrowheads depict c-Jun-positive perigranulomatous hepatocyte nuclei in *S.* *mansoni*-infected c-Jun^f/f^ control mice, blue arrowheads: c-Jun-negative perigranulomatous hepatocyte nuclei in *S.* *mansoni*-infected c-Jun^Δli^-mice, red arrows: c-Jun-positive nuclei of non-parenchyma cells, *: *S. mansoni* egg, dashed line indicates granuloma; scale bars 100 μm, magnification x 200. Please note the schematic illustration of the major histologic outcome of the hepatocyte specific knockout of c-Jun below representative micrographs. The microphotographs of the immunostainings of Fig. 1B are presented in enlarged form in SFig. 1. (**C**) Western blot analysis and subsequent densitometric assessment showed enhanced expression of c-Jun in infected animals (*S.m.*) compared to non-infected controls (ni) and enhanced expression of c-Jun in infected c-Jun^f/f^ animals (f/f *S.m.*) compared to infected c-Jun^Δli^ animals (Δli *S.m.*). n = 6 and 3 technical replicates. The indicated *p* values were calculated by ANOVA and post hoc pairwise comparison of groups using Fisher’s LSD on log transformed data. Schematic illustrations were created with BioRender.com.

To validate a successful and hepatocyte-specific knockout of c-Jun in the mouse model for *S.* *mansoni* infection (Fig. 1a), immunohistochemical analysis with specific c-Jun antibodies was performed. The infection with *S.* *mansoni* induced c-Jun expression in perigranulomatous hepatocytes (Fig. 1b). Floxed c-Jun alleles in c-Jun^f/f^ mice did not interfere with c-Jun expression in perigranulomatous hepatocytes, indicated by c-Jun positive hepatocyte nuclei (Fig. 1b, red arrowheads). In contrast, hepatocyte nuclei in c-Jun^Δli^ mice were negative for c-Jun staining (Fig. 1b, blue arrowheads). Furthermore, it was demonstrated that other hepatic cells, except hepatocytes, in c-Jun^Δli^ mice, were still able to express c-Jun to a similar extent as in c-Jun^f/f^ mice (Fig. 1b, enlarged in SFig. 1, red arrows). This indicated a successful hepatocyte-specific knockout of c-Jun in c-Jun^Δli^ mice (Fig. 1b). Please note the explanatory scheme depicting the most important effects in the lower part of Fig. 1b. Western blotting showed an increased hepatic c-Jun expression upon *S.* *mansoni* infection for both genotypes. Nevertheless, the amount of c-Jun quantified by optical densitometry was lower in c-Jun^Δli^ mice compared to c-Jun^f/f^ mice, reflecting that only non-hepatocyte liver cells were able to express c-Jun in the liver of c-Jun^Δli^ mice (Fig. 1c).

### Upon *S.* *mansoni* infection, no changes in fibrogenesis but enhanced hepatic damage occurred in mice with hepatocyte-specific knockout of c-Jun

**Figure 2:**
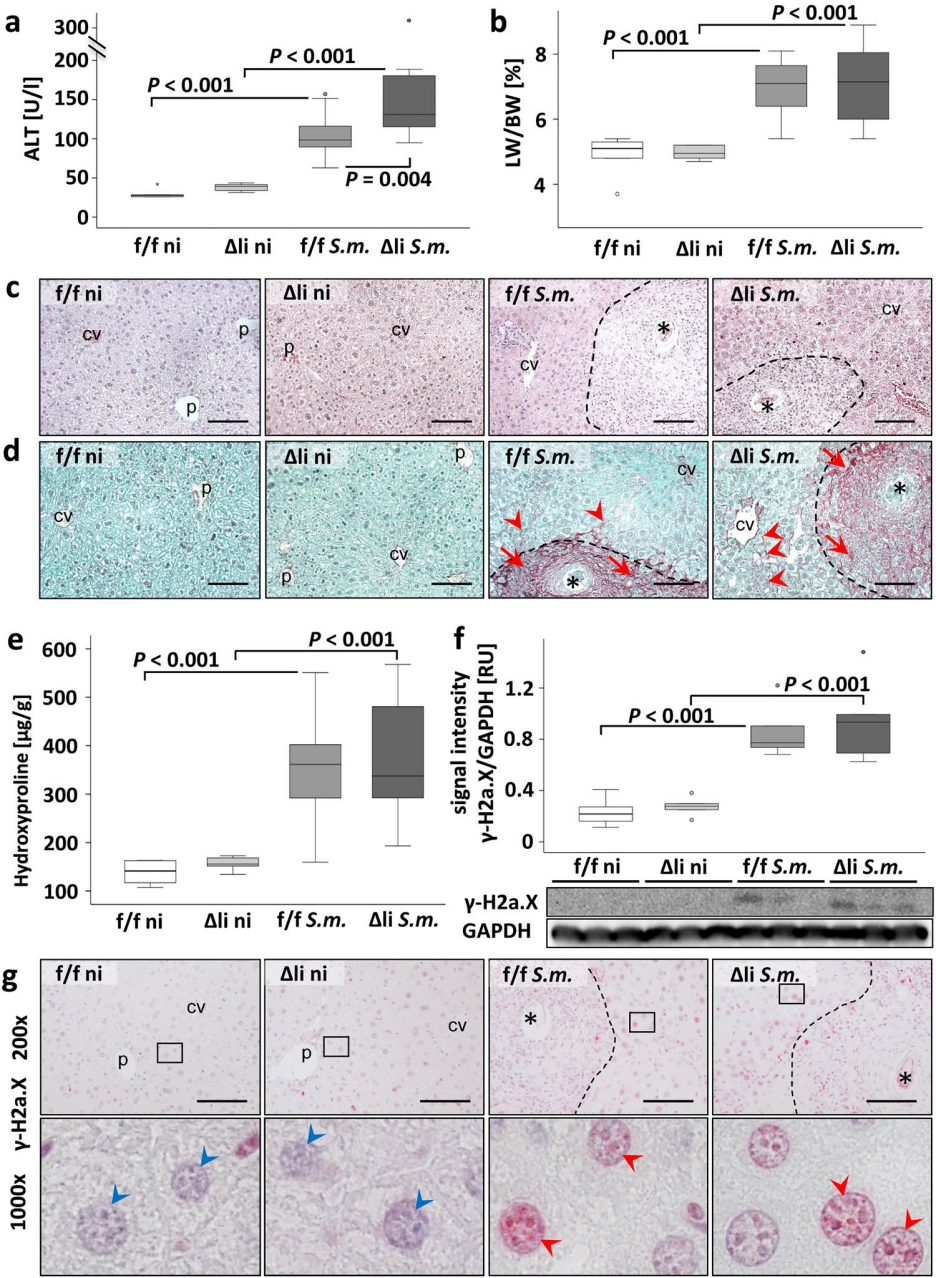
Serum markers for liver damage were enhanced in *S.* *mansoni*-infected c-Jun^Δli^ mice. (**A**) ALT concentration was elevated in infected animals and ALT levels were even higher in infected c-Jun^Δli^ animals compared to infected c- Jun^f/f^ animals. n = 6 f./f ni, n = 6 Δli ni, n = 10 f./f *S.m.*, n = 12 Δli *S.m.,* including 2 technical replicates each. (**B**) Liver weight/ body weight ratio was elevated in infected animals. n = 6 f./f ni, n = 6 Δli ni, n = 11 f./f *S.m.*, n = 12 Δli *S.m.* (**C**) H.E.-staining visualized hepatic granuloma formation induced by the *S.* *mansoni* eggs. No differences between the infected animals were detected (enlarged microphotographs depicted in SFig. 3). (**D**) Sirius-red staining visualized granulomatous fibrosis (red arrows), and sinusoidal (red arrowheads) fibrosis (for details please refer to enlarged microphotographs depicted in SFig. 5). We detected no differences in histopathologic appearance of fibrosis between the infected animals. *S.* *mansoni* eggs (*). cv central vein, p portal tract, 200 x, bar 100 µm, dashed lines indicate granuloma. (**E**) Hydroxyproline quantification indicated enhanced fibrosis in the liver of infected animals but no quantitative differences in hepatic amounts of fibrillary collagen between the two infected groups. (**F**) Western blot analysis of γ-H2a.X and subsequent assessment of optical density of the signals depicted an elevation of the marker for DNA double-strand breaks in the infected groups compared to non-infected animals but showed no difference between the infected animals. n = 6 and 3 technical replicates each. (G) Immunohistochemical staining of γ-H2a.X visualized an elevated amount of γ-H2a.X in nuclei of hepatocytes of infected animals. Lower panels show the indicated areas with higher magnification. Enlarged microphotographs are shown in SFig. 6. Red arrowheads point to γ-H2a.X positive nuclei of hepatocytes, blue arrowheads indicate γ-H2a.X-negative nuclei of hepatocytes. 200 x, scale bars 100 µm. The indicated *p* values were calculated by ANOVA and post hoc pairwise comparison of groups using Fisher’s LSD on log transformed data.

To assess liver damage, multiple tests were performed to gather information whether c-Jun expression in hepatocytes is crucial for hepatoprotection during *S.* *mansoni* infection. The hepatocyte-specific serum marker alanine transaminase (ALT) (Fig. 2a) and aspartate transaminase (AST) (SFig. 2a) were increased in mice upon *S.* *mansoni* infection. Additionally, the induction of ALT and AST was more prominent in infected c-Jun^Δli^ mice compared to infected c-Jun^f/f^ mice. The liver weight/ body weight ratio was also elevated in *S.* *mansoni*–infected mice, but no differences were detected between the two infected animal groups (Fig. 2b). The hepatic egg load, determined by potassium-hydroxide digestion, was equal in both groups of infected animals (SFig. 2b). H&E staining visualized the granulomatous hepatic pathology induced by the *S.* *mansoni* infection (Fig. 2c, enlarged in SFig. 3). The livers of infected animals showed no visible differences in histopathologic changes depending on the genotype, and neither the number of granulomas nor their size differed between the two infected animal groups (Fig. 2c, SFigs. 3 and 4).

All non-infected animals displayed a healthy liver histology with no abnormalities (Fig. 2c). Sirius-red staining visualized hepatic fibrosis induced by *S.* *mansoni* eggs (Fig. 2d, enlarged in SFig. 5). No fibrosis was detected in the livers of non-infected control animals. Noteworthy is the pattern of fibrosis in infected animals. Fibrillar collagen is mainly located within granulomas around the eggs (red arrows) with small amounts of sinusoidal fibrosis (Fig. 2d, red arrowheads). To quantify the amount of fibrosis in the liver, a hydroxyproline assay was performed. Quantification of hepatic hydroxyproline indicated an elevated amount of fibrillar collagen in the liver of infected animals and equal amounts in the two groups of infected animals (Fig. 2e). To analyze hepatic DNA-double strand breaks, induced by *S.* *mansoni* infection^20^, western blotting and immunohistochemistry for the marker γ-H2a.X were performed. γ-H2a.X was induced upon *S.* *mansoni* infection and comparable signal intensities were observed in infected animals regardless of their phenotype (Fig. 2f). Perigranulomatous hepatocyte nuclei of infected animals were positively stained for γ-H2a.X, whereas the hepatocyte nuclei in non-infected animals were not stained (Fig. 2g, enlarged in SFig. 6).

### Markers for hepatocellular stress were not regulated upon c-Jun knockout in *S.* *mansoni*-infected mice

**Figure 3:**
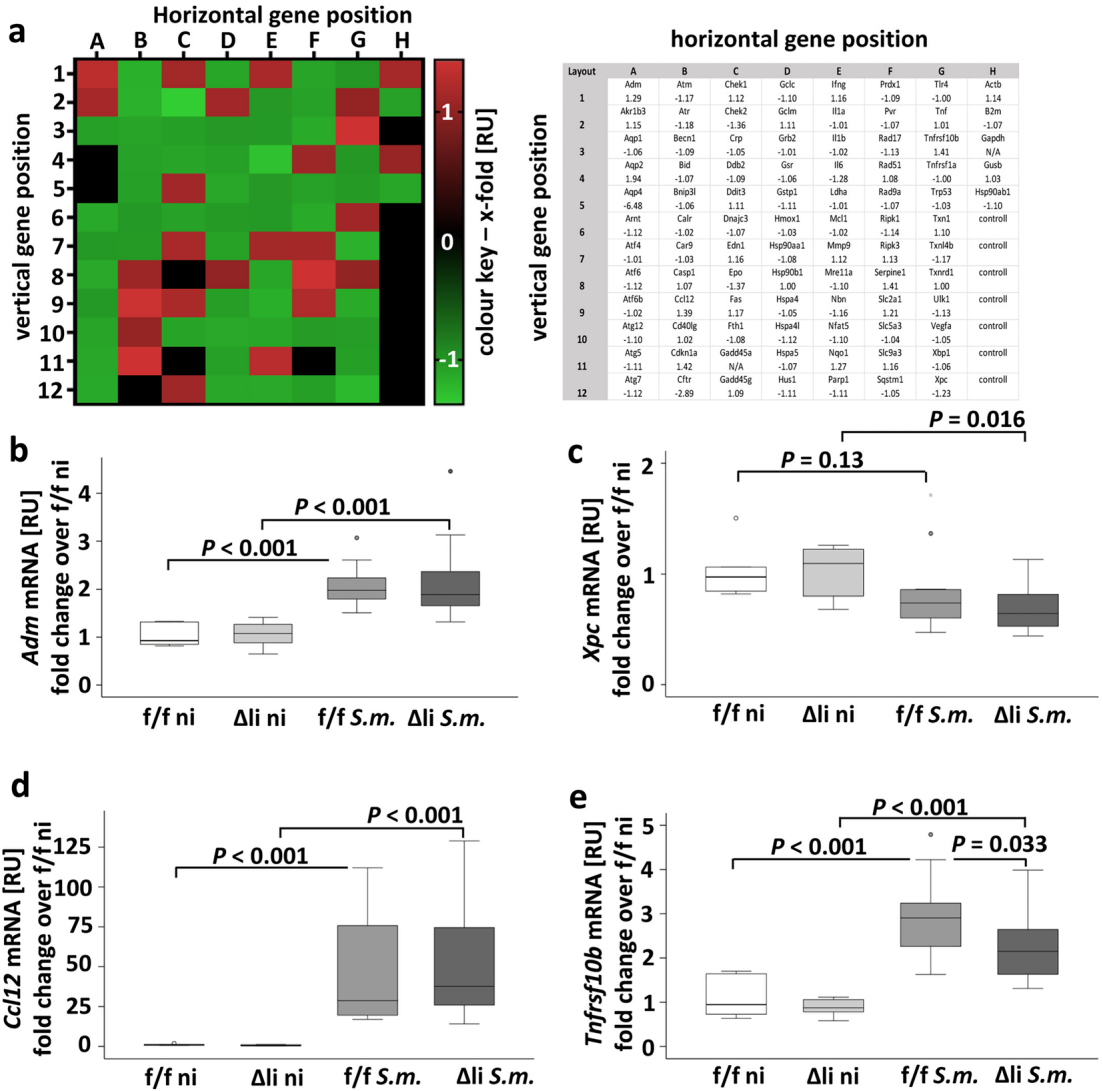
qPCR array and subsequent RT-qPCR validation of marker genes for hepatocellular stress and toxicity. (**A**) 84 genes related to stress and toxicity were analyzed by a qPCR array. The signal for each gene was normalized with housekeeping genes, and respective x-fold values from infected c Jun^f/f^ and infected c-Jun^Δli^ animals were used to draw the heat map. Reddish tone indicates an induction while green shades depict downregulated genes as indicated by the colour key on the right of the heatmap. Ct-values above 30 are displayed as black panels in the heat-map. Gene names on positions A1-H12 and their specific regulation are presented in the table on the right side of (**A**). (**B**–**E**) Genes regulated above 1.2-fold in the array were validated by RT-qPCR. (**B**, **D**, **E**) *Adm*, *Ccl12* and *Tnfrsf10b* were induced in infected animals. Please note the reduction of *Tnfrsf10b* and *Xpc* in Δli *S.m*. (**C**) n = 6 f./f ni, n = 6 Δli ni, n = 11 f./f *S.m*., n = 12 Δli *S.m*., 3 technical replicates. The indicated *p* values were calculated by ANOVA and post hoc pairwise comparison of groups using Fisher’s LSD on log transformed data.

To receive a profound overview of presumed hepatocellular stress occurring in *S.* *mansoni*-infected animals, a PCR array addressing 84 genes associated with oxidative stress, hypoxia signaling, osmotic stress, cell death, inflammatory response, unfolded protein response, and DNA damage and repair, was performed (Fig. 3a). Genes that were > 1.2-fold up- or downregulated were validated by RT-qPCR, i.e. *Adm, Xpc, Ccl12, Tnfrsf10b, Chek2*, and *Cdkn1a*. RT-qPCR confirmed an upregulation for *Adm, Ccl12, Tnfrsf10b*, and *Cdkn1A* upon *S.* *mansoni* infection (Fig. 3b–e and SFig. 7). The expression of *Xpc* was slightly reduced in infected c-Jun^Δli^ mice but not in c-Jun^f/f^ mice (Fig. 3c). Besides a slight reduction of *Tnfrsf10b* (Fig. 3e), no differences in any of the analyzed genes were detected between the two infected animal groups (Fig. 3B–E, SFig. 7a+b+b).

Because c-Jun has also been shown to play a pivotal role in resolving oxidative stress^18^, western blotting was performed to detect catalase, a marker for oxidative stress. A downregulation of catalase was evident upon *S.* *mansoni* infection, with equal levels in infected c-Jun^Δli^ and infected c-Jun^f/f^ mice (SFig. 8a). Additionally, an assay to quantify malondialdehyde (MDA) was performed, an established method to quantify oxidative stress by the measurement of lipid peroxidation^21^. The amount of MDA was similar in all mice (SFig. 8b). As c-Jun is involved in ER stress and the unfolded protein response^17^, prominent ER-stress markers^22^ like *Bip, Chop,* and *Xbp1s* were analyzed by RT-qPCR (SFig. 8c–e). In addition, BiP, eIF2a, and p-eIF2a were analyzed by western blotting (SFig. 8f). While eIF2a, p-eIF2a, BiP, and Chop showed no regulation in any group, *Xbp1s* expression was downregulated upon infection compared to the non-infected animals (SFig. 8C–F). However, *Xbp1s* expression levels were also evenly reduced in the two infected animal groups. *S.* *mansoni* infection, cell stress and altered metabolism often lead to impaired autophagy, which could also be the cause of increased cell death^23^. As c-Jun can link autophagy with cell survival^17^, another PCR-array was performed with 84 genes involved in autophagy. No relevant changes were detected in c-Jun^Δli^ mice compared to infected c-Jun^f/f^ mice in this array as indicated by the moderately stained heatmap and the individually depicted results in the associated table (SFig. 9).

### The induction of hepatic inflammation in *S. mansoni*-infected mice was independent of c-Jun expression in hepatocytes

**Figure 4:**
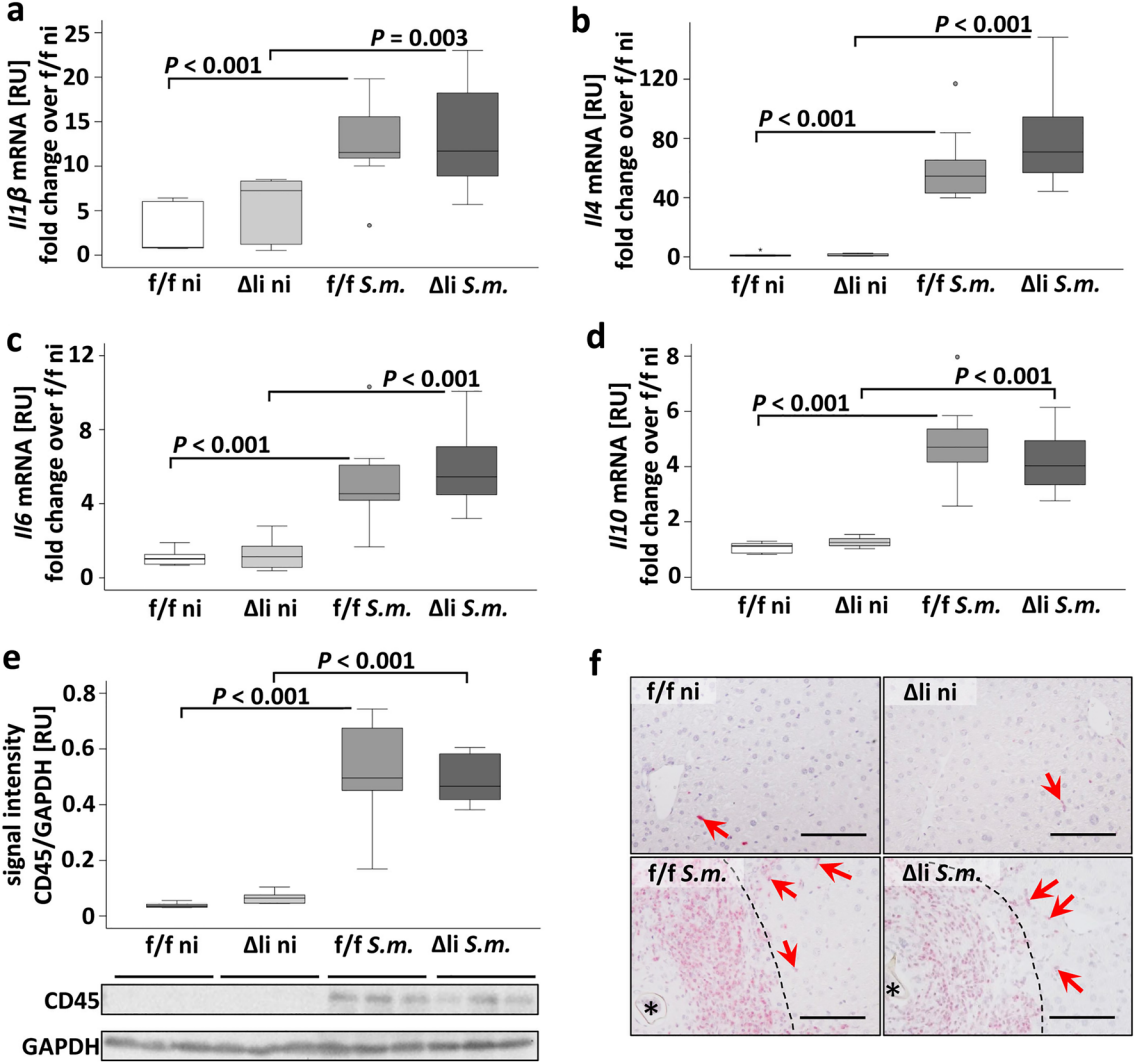
Hepatic inflammation was induced by *S.* *mansoni* infection and equally regulated in groups of infected c-Jun^f/f^ and infected c-Jun^Δli^ animals. (**A**–**D**) RT-qPCR demonstrated the induction of *Il-1β*, *Il-4*, *Il-6*, and *Il-10*, in infected animals compared to non-infected animals, but no differences among infected or ni animals. n = 6 f./f ni, n = 6 Δli ni, n = 11 f./f *S.m*., n = 12 Δli *S.m*.; 3 technical replicates each. (**E**) Western blot analysis and subsequent assessment of optical density of the signals depicted elevated expression for CD45, a marker for leukocytes, in infected animals compared to non-infected animals. n = 6 and 3 technical replicates each. (**F**) CD45 staining demonstrated CD45-positive granulomatous cells and CD45-positive cells within the parenchyma (red arrows, enlarged in SFig. 11). Both analyses of CD45 expression showed a comparable expression pattern of CD45 in infected c-Jun^Δli^ and infected c-Jun^f/f^ mice. Magnification 1000 × , bars 100 µm, dashed line granuloma. The indicated *p* values were calculated by ANOVA and post hoc pairwise comparison of groups using Fisher’s LSD on log transformed data.

Hepatic inflammation was assessed by different methods to investigate whether a lack of c-Jun expression in hepatocytes affects inflammation. The immune reaction against *S.* *mansoni* starts with a T-helper-1 (TH1) response in the first weeks after infection. This turns into a T-helper-2 (TH2) response to fight the parasite in the different stages of the hepatic pathogenesis^23^. The TH1-specific cytokines *Il-1β, Tnf-α, Ifn-γ,* the TH2 specific *Il-4*, and other inflammatory cytokines such as *Il-6* and *Il-10* were induced upon *S. mansoni* infection (Fig. 4a–d and SFig. 10).

The respective extent of cytokine activation appeared to be equivalent in the infected groups. (Fig. 4a–d, SFig. 10a+b). Hepatic expression of the pan-leukocyte marker CD45 was enhanced upon *S.* *mansoni* infection. The induction of CD45 was similar in the infected animal groups (Fig. 4e). Immunohistochemistry of CD45 demonstrated the vast and concentrated immunological reaction surrounding the eggs (Fig. 4f, enlarged in SFig. 11). CD45-positive cells also infiltrated parenchymal tissue, but hepatic inflammation was mainly characterized by granuloma formation around the eggs. Just a few CD45 positive cells appeared in the hepatic tissue of non-infected animals.

### Following *S.* *mansoni* infection, hepatocyte proliferation was reduced by c-Jun knockout

**Figure 5:**
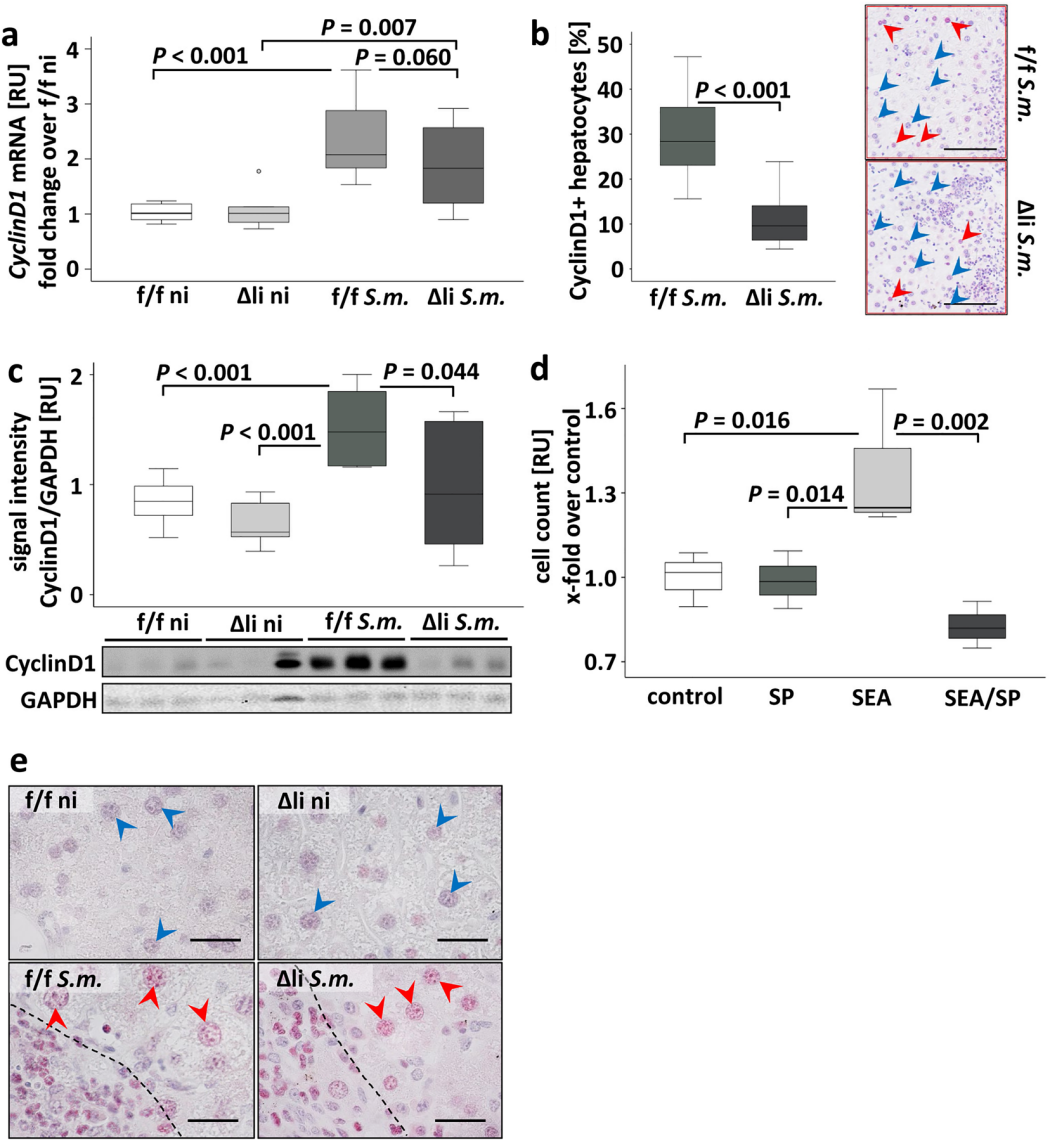
*S. mansoni* soluble egg antigen induced proliferation of hepatocytes was diminished by inhibition or knockout of c-Jun. (**A**) C*yclin-D1* was induced upon *S.* *mansoni* infection in comparison to non-infected animals. A tendency of a lower expression of *Cyclin D1* was evident in Δli *S.m*. compared to f/f *S.m..* n = 6 f./f ni, n = 6 Δli ni, n = 11 f./f *S.m*., n = 12 Δli *S.m*.; 3 technical replicates. (**B**) The relative number of Cyclin D1-positive perigranulomatous hepatocyte nuclei was lower in *S.* *mansoni*-infected Δli *S.m.* mice. Representative microphotographs of immunostained liver slices are shown. Hepatocyte nuclei in three randomly chosen areas per mouse adjacent to granuloma were counted (red arrowheads Cyclin D1-positive hepatocyte nuclei, blue arrowheads Cyclin D1 negative hepatocyte nuclei; microphotographs enlarged in SFig. 14). (**C**) Western blot analysis and subsequent assessment of optical density of the signals demonstrated an induction of hepatic Cyclin D1 upon *S.* *mansoni* infection. This effect was normalized in Δli *S.m*. compared to f/f *S.m.* (n = 6 and 3 technical replicates each)*.* (**D**) Proliferation of human hepatoma cells increased by *S.* *mansoni* soluble egg antigen (SEA) treatment and remained at basal levels by the addition of JNK inhibitor SP600125. Data were normalized to the control group. These experiments were performed three times independently. Levels of significance are indicated in the figure. (**E**) Ki67 immunostaining demonstrated proliferating hepatocytes upon infection. Ki67-positive nuclei in perigranulomatous hepatocytes (red arrowheads) and Ki67 negative nuclei in non-infected animals (enlarged microphotographs of the Ki67 immunostainings are depicted in SFig. 15).

As c-Jun represents an essential modulator and initiator of liver regeneration and hepatocyte proliferation^15,16^, we analyzed the hepatic expression of different proliferation markers^24^. Western blot analysis of Proliferating Cell Nuclear Antigen (PCNA), which controls DNA replication, showed an elevated protein expression in infected animals but no group differences among infected animals (SFig. 12). *Mcm2* and *Cyclin D1* were amplified in c-Jun^f/f^ mice as well as in c-Jun^Δli^ mice upon infection. While the hepatic level of *Mcm2* was not regulated between the groups of infected animals (SFig. 13), which might be due to the strong hepatic infiltration of inflammatory cells, *Cyclin D1*-induction tends to be lower (*P* = 0.060) in infected c-Jun^Δli^ mice compared to infected c-Jun^f/f^ mice (Fig. 5a). As *Cyclin D1* mRNA levels were assessed as a measure over all liver cells, the hepatocyte-specific effect may be masked by the regulation of *Cyclin D1* in infiltrating inflammatory cells. Therefore, we counted Cyclin D1-positive perigranulomatous hepatocyte nuclei in randomly chosen perigranulomatous areas of positively immunostained liver slices (Fig. 5b and SFig. 14). Interestingly, the relative number of Cyclin D1-positive perigranulomatous hepatocyte nuclei was lower in *S.* *mansoni*-infected c-Jun^Δli^ mice (Fig. 5b, microphotographs of immunostained liver slices are depicted enlarged in SFig. 14). Please note in SFig. 14 that considerable numbers of cells inside the granuloma were stained positive for Cyclin D1. To complete the picture, we also assessed hepatic protein expression of Cyclin D1 by western blotting (Fig. 5c). *S.* *mansoni* induced hepatic Cyclin D1 was lower in infected c-Jun^Δli^ mice. It has recently been suggested that *S.* *mansoni* soluble egg antigens induce hepatocyte proliferation^25^. In order to define the hepatocyte specific effect of c-Jun deactivation on proliferation, we inhibited JNK/c-Jun signaling using the inhibitor SP600125 in soluble egg antigen (SEA)-stimulated human hepatoma cells (HepG2) and assessed the cell count. Figure 5d shows the relative cell count in comparison to the control group. *S.* *mansoni* SEA-induced proliferation of human hepatoma cells was normalized by inhibition of JNK/c-Jun-signaling (SP).

Immunohistochemical staining of Ki67 showed positive hepatocyte nuclei in the infected animals. Close to the granuloma almost all hepatocytes were positive for Ki67, whereas with increasing distance to the granuloma the rate of Ki67-positive hepatocyte nuclei decreased. Non-infected control animals showed almost no Ki67 positive hepatocytes (Fig. 5e, enlarged in SFig. 15). Please note that almost all nuclei of cells inside the granulomas were stained positive for Ki67.

As hepatic glutaminase 2 (*Gls2*) is regulated by c-Jun and may be involved in cell proliferation^26^ we analyzed *Gls2* by RT-qPCR.The expression of *Gls2* was reduced upon *S. mansoni* infection, and equal expression was detected in the groups of infected animals, independent of c-Jun expression (SFig. 16). The reduced hepatic expression of *Gls2* caused by *S.* *mansoni* infection may be caused by the metabolic exhaustion of the liver, as we described recently^27^.

## Discussion

The current study addressed the question, whether c-Jun has a hepatoprotective and regenerative role in *S.* *mansoni* infection. Indeed, we demonstrated that a hepatocyte-specific c-Jun knockout increased hepatocellular damage in infected mice. The elevated serum levels of ALT and AST in c-Jun^Δli^ mice upon *S.* *mansoni* infection indicate its anti-inflammatory and hepatocyte-protective role in our model system. Nevertheless, none of the analyzed c-Jun-dependent stress responses for hepatocellular damage gave a compelling explanation for the elevated aminotransferase levels in *S.* *mansoni* infected c-Jun^Δli^ mice. However, a reduced regenerative potential of injured hepatocytes in infected c-Jun^Δli^ mice is reflected by Cyclin D1, which was significantly induced in *S.* *mansoni*-infected c-Jun^f/f^-mice and c-Jun^Δli^ mice but with a higher expression in c-Jun^f/f^ mice (Fig. 5b and c). In the first postnatal weeks, the liver expands remarkably to reach the adult size^28^, and embryonic lethality of global c-Jun knockout mice is attributable to the important role of c-Jun in hepatocyte proliferation^29^. Furthermore, increased cell death and decreased hepatocyte proliferation after hepatectomy in the absence of hepatocellular c-Jun has been demonstrated before^15^. In addition to the lower degree of hepatic *Cyclin D1* induction in *S.* *mansoni*-infected c-Jun^Δli^ mice, the more pronounced expression of CDK-inhibitor 1 (*Cdkn1a*) might also reflect a reduced regenerative potential of injured hepatocytes in mice lacking hepatocellular c-Jun. Of note, we recently described that *S.* *mansoni* SEA triggered the cell cycle in hepatocytes and promoted proliferation^25^. In order to analyze if *S.* *mansoni* SEA-triggered proliferation of hepatocytes depends on JNK/c-Jun signaling, we simulated the situation in cell culture. Compelling, *S.* *mansoni* SEA-induced proliferation of human HepG2 cells was normalized by the addition of the specific JNK inhibitor SP600125 (Fig. 5d).

Serum ALT represents a specific and sensitive marker for hepatocyte damage. Serum ALT levels can be elevated due to infections, alcohol consumption, medication, autoimmune diseases, and dietary habits^30^. Hence, hepatocyte damage results in ALT released into the bloodstream, which can be measured in the serum^30^. Therefore, we conclude an elevation in hepatocyte damage under hepatocellular c-Jun knockout conditions in *S.* *mansoni*-infected mice. Nevertheless, during *S. mansoni* infection, the hepatocyte damage is relatively moderate with about 120 U/l compared to other liver infection models. In viral hepatitis infections, transaminases might be elevated up to 1,000 U/l^31^. When the impact of c-Jun in hepatocytes was investigated in other mouse models, usually more aggressive liver injuries were observed, and the difference between c-Jun knockout animals and controls were found to be more extensive in terms of ALT- and AST-levels^17,18^. It should be considered that differences in the underlying biomolecular causes of a pathological effect might be more difficult to detect, if the disease pattern is less severe. The high spread of egg load per mg of liver tissue (SFig. 1) could enhance this effect, since hepatic pathology is essentially caused by the eggs of the parasite. Nevertheless, the accumulation of smaller effects like the slight reduction of *Tnfrsf10b* and *Xpc* (Fig. 3C, E), the trending increased signal for γ-H2a.X (Fig. 2f) or IL-4 (Fig. 4b) could also contribute to enhanced liver damage in c-Jun^Δli^ mice.

Another important aspect is that other hepatocellular signaling pathways with redundant cellular functions may be able to effectively compensate for the moderate effects of the c-Jun knockout in c-Jun^Δli^ mice. Recently, we demonstrated that STAT3 signaling is also activated upon *S. mansoni* infection^20^. Since both factors, c-Jun and STAT3, are involved in a variety of homologous hepatocellular functions, e.g. in the stress response in the acute phase^32^, in metabolism^33^, or in proliferation^34^, compensation for the loss of c-Jun seems likely.

In tests such as the assessment of fibrosis or measurement of inflammation, it must be taken into account that the c-Jun knockout is hepatocyte-specific, while other liver cells such as fibroblasts or immune cells are still able to express c-Jun to a normal extent. Since these cells are not affected by the knockout, they respond normally to *S.* *mansoni* infection. Collagen production or immune cell invasion might not be affected by the hepatocyte-specific knockout and thus are not directly related to the hepatocellular damage we observed. Since the analysis was performed in whole liver sample, the effect of c-Jun knockout in hepatocytes can be masked by the other liver or liver-infiltrating cells. Therefore, we performed immunohistochemical analyses to visualize cell type-specific responses. The results supported our observation that the effects were hepatocyte-specific. However, none of the processes analyzed appeared to be solely responsible for the exacerbation of hepatocellular damage in c-Jun^Δli^ mice.

The overall picture suggests that many different processes may have caused, in combination, the increased hepatocyte damage in c-Jun knockout mice. Another important point to consider is the duration of the experimental mouse infection with *S.* *mansoni*, which allowed us to analyze one given point of the pathologic chronification of schistosomal hepatitis*.* The experimental infection was stopped after nine weeks, when the mice were euthanized and the organs prepared for further research. At this point, the acute phase of infection is turning over into the chronic phase, and the progressing pathology gets more severe from this time point on^35^. It may be speculated, that c-Jun would have a more pronounced effect in later phases of the infection. However, with respect to 3R principles, a higher number of animals per group and later time points for euthanasia were not considered to avoid additional pain or fatal outcomes due to severe liver damage^36^. The slight tendential increase in ALT levels in uninfected c Jun^Δli^ mice compared to uninfected c-Jun^f/f^ mice suggested a naturally higher ALT concentration in the serum of c-Jun^Δli^ mice due to the knockout. The infection with schistosomes might magnify this basal difference in ALT. In this case, c-Jun would provide an ALT-independent additional protection during infection.

It was recently suggested, that the decrease in hepatocyte division observed in liver disease may not only be a consequence of fibrosis and inflammation, but could also be an etiology of liver pathology^37^. The authors investigated, whether blocking hepatocyte division in a mouse model affects physiology or clinical liver manifestations. This model displayed many of the hallmarks that are found in human patients with liver disease, e.g. enhanced serum ALT, inflammation, and fibrosis. Most strikingly, the phenotypes of this model developed without any external insults^37^. In regard to this, it seems likely, that the regression of the regenerative capacity of hepatocytes by c-Jun knockout in *S.* *mansoni* infected mice is at least one cause for enhanced hepatocyte damage displayed by the raise of serum ALT.

Limitation: An infection with fifty to one hundred *S.* *mansoni* cercariae per mouse represents the usual amount of cercariae used for experimental schistosomiasis^38^. The amount of worm couples per infected mouse is not comparable to the human infection, since the most serious infection detected in an autopsy in men rarely exceeded five worm-pairs per kilogram of body weight^38^. Nevertheless, the use of 50–100 cercariae per mouse induces a disease resembling the human disorder^38^. Therefore, experimental schistosomiasis as described herein was performed with considerably high numbers of cercariae to provoke severe clinical patterns within a defined time scale.

**Figure 6:**
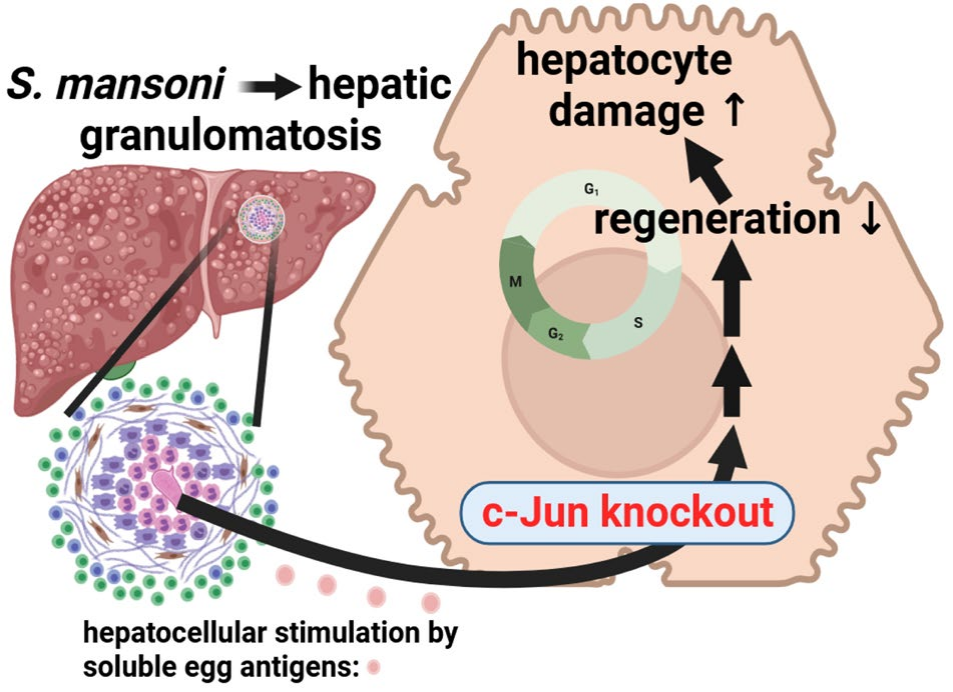
Schematic summary of the most important results. The current study underlines the hepatoprotective role of c-Jun in *S.* *mansoni*-induced liver damage. Our data suggest that the c-Jun-controlled regenerative hepatocellular potential is involved in cellular protection via c-Jun signaling.

In summary, our results suggest a protective effect of c-Jun on hepatocyte integrity in mouse livers of a schistosome infection model. This protective mechanism may be based on preserved cell cycle control and improved regeneration (schematically summarized in Fig. 6). Although further studies are needed to pinpoint the molecular mechanism of c-Jun´s positive effect in this physiological scenario, our findings delivered further insights into the c-Jun network, and how hepatocytes behave under c-Jun knockout conditions in an infectious disease context. Despite the fact that the protective effect of c-Jun in *S.* *mansoni* infection was not as clear as in other models of liver injury, the direction in which c-Jun provides hepatocellular protection seems to be similar, and provided further evidence for the central role of c-Jun in cell survival and death.

## Materials and methods

### Animal model

*Biomphalaria glabrata* snails were used as intermediate hosts for maintaining the *S.* *mansoni* life cycle, and C57BL/6 mice were used as final hosts. Mice with conditional alleles of c-Jun (c-Jun^f/f^) were provided by the inventor on a mixed genetic background (C57BL/6 × 129/Sv)^15^ and crossed back to C57BL/6 J for five generations. c-Jun^f/f^ mice were crossed with transgenic AlbCre mice^39^ (RRID:IMSR_JAX:003574) to obtain animals with hepatocyte-specific knockout of c-Jun (c-Jun^Δli^). Corresponding c-Jun^f/f^ mice expressing c-Jun but without Cre recombinase transgene were used as controls. At the age of eight weeks, twelve randomly chosen c-Jun^Δli^ mice and twelve c-Jun^f/f^ mice were infected with 100 cercariae (both sexes) in a water bath tempered to 30 °C using the pre-soaking procedure that was described before^40^. Six randomly chosen non-infected c-Jun^Δli^ mice and six non-infected c-Jun^f/f^ mice were used as super controls. At the age of 17 weeks, the mice were anesthetized with Isofluran (4% v/v, ecuphar GmbH), subsequently euthanized by cervical dislocation, and the organs were used for further analysis. Liver samples were shock frosted and stored at − 80 °C or preserved for histology as indicated below. Serum samples were stored at − 80 °C until analysis of aminotransferases by routine clinical chemistry on a Reflotron Plus Analyzer (Roche, Mannheim, Germany). The success of the infection was validated by evidence of eggs in stool and liver of the mice. Stool and liver of one of the c-Junf/f controls, which were treated with *S. mansoni* cercariae, were free of parasitic eggs. This mouse was not considered for further analysis. The study is reported in accordance with ARRIVE guidelines^41^. All animal experiments have been done in accordance with the European Convention for the Protection of Vertebrate Animals used for experimental and other scientific purposes (ETS No 123; revised Appendix A) and were approved by the Regional Council Giessen (V 54 - 19 c 20 15 h 01 GI 20/10 Nr. G 44/2019).

### Western blot analysis

Protein analysis was performed as described previously^20^. Primary antibodies used were (Dilution 1:1000 in 5% BSA): c-Jun Cell Signaling (#9165), γ-H2a.X Cell Signaling (#80312S), CD45 Cell Signaling (#72787), PCNA Cell Signaling (#13110), Catalase Gene Tex (GTX110704), BiP Cell Signaling (#3177), eIF2a Cell Signaling (#5324P), p-eIF2a Cell Signaling (#9721), GAPDH Proteintech (60004-1-Ig). Secondary antibodies used were (Dilution 1:5000 in 5% dry milk): Goat anti rabbit antibody Cell Signaling (#7074), Horse anti mouse antibody Cell Signaling (#7076).

### Cell culture experiments

HepG2 cells (stock ordered in 2019, CLS # 330198, expanded and stored as cryostocks for consistent quality in culture for up to 10 passages per cryostock) were stimulated after 24 h fasting in DMEM w/o FCS with 15 µg/ml SEA and/or 10 µM JNK-inhibitor SP600125 for 4 h.

### Immunohistochemistry and Sirius-red staining

Immunohistochemistry (IHC) was performed with 3 µm formalin-fixed and paraffin-embedded liver sections with ImmPRESS AP REAGENT KIT (MP-5401) from Vector Laboratories, Inc. (California, USA). Deparaffination, unmasking, and blocking was performed as described before^42^. Primary antibodies used were: c-Jun Cell Signaling (#9165), γ-H2a.X Abcam (ab81299), CD45 Cell Signaling (#70257), KI67 LSBio (LS‑B13463). Color reaction was developed with Permanent AP Red Kit (ZYT-ZUC001-125) from Zytomed Systems (Berlin, Germany).

Sirius-red staining was performed as described in^43^.

### PCR array

RT^2^ Profiler PCR arrays from Qiagen (Qiagen N.V., Venlo, Netherlands) were performed according to manufacturer's protocol with mouse liver samples (Mouse Stress & Toxicity PathwayFinder (330231 - PAMM-003Z) and Mouse Autophagy (330231 - PAMM-084Z).

### Quantitative real-time PCR

The isolation of mRNA was performed with RNeasy® Mini Kit from Qiagen (Cat. No. 74106) according to the manufacturer’s protocol, and cDNA synthesis was performed with iScript cDNA Synthesis Kit from Bio-Rad (Cat. No. #1708891) (Bio-Rad Laboratories, Inc., Hercules, California, USA) according to manufacturer’s protocol. RT-qPCR was performed as described recently^44^. Primers used were:

*Adm* (sense: 5′-agc tgg ttt cca tca ccc tg-3′, antisense: 5′-tct cat cag cga gtc ccg ta-3′), *Xpc* (sense: 5′-agg cgg tgg aga ttg aaa ttg-3′, antisense: 5′-cag gtg aac ctt gtg cat gtt-3′), *Ccl12* (sense: 5′-att tcc aca ctt cta tgc ctc ct-3′, antisense: 5′-atc cag tat ggt cct gaa gat ca-3′), *Tnfrsf10b* (sense: 5′-agt gtg tct cca aaa cgg ct-3′, antisense: 5′-cag agt tcg cac ttt cgg ga-3′), *Il-1β* (sense: 5′ –tga cag tga tga gaa tga cct g- 3′, antisense: 5′ –cgg gaa aga cac agg tag ct- 3′), *Il-4* (sense: 5′-ggt ctc aac ccc cag cta gt-3′, antisense: 5′-gcc gat gat ctc tct caa gtg at-3′), *Il-6* (sense: 5′-tcc agt tgc ctt ctt ggg ac-3′, antisense: 5′-gta ctc cag aag acc aga gg-3′), *Il-10* (sense: 5′-ccc att cct cgt cac gat ctc-3′, antisense: 5′-tca gac tgg ttt ggg ata ggt tt-3′), *Cyclin D1* (sense: 5′-gcg tac cct gac acc aat ctc-3′, antisense: 5′-ctc ctc ttc gca ctt ctg ctc-3′), *Mcm2* (Cat. No.: QT00110348, QuantiTect Primer Assay), *Chek2* (sense: 5′-gat cat tag caa gcg gag gtt -3′, antisense: 5′-cac cac ccg gtc aaa tag ttc-3′), *Cdkn1a* (sense: 5′-cct ggt gat gtc cga cct g-3′, antisense: 5′-cca tga gcg cat cgc aat c-3′), *Bip* (Cat. No.: QT00172361, QuantiTect Primer Assay), C*hop* (sense: 5′-ctg gaa gcc tgg tat gag gat-3′, antisense: 5′-cag ggt caa gag tag tga agg t-3′), *Xbp1s* (5′-gag tcc gca gca ggt g-3′:, 5′-gtg tca gag tcc atg gga-3′:), *Tnf-α* (sense: 5′ –gcc cac gtc gta gca aac cac- 3′, antisense: 5′ –gca ggg gct ctt gac ggc ag- 3′), *Ifn-γ* (sense: 5′ -ctg cat ctt ggc ttt gca gc- 3′, antisense: 5′ -aga taa tct ggc tct gca gga t- 3′), *β-Actin* (sense: 5′-ggc tgt att ccc ctc cat cg-3′, antisense: 5′-cca gtt ggt aac aat gcc atg t-3′), *Gls2* (sense: 5′-cgt ccg gta cta cct cgg t-3′, antisense: 5′-tgt ccc tct gca ata gtg tag aa-3′).

### Serum analysis

The serum parameters ALT and AST were analyzed by Roche Reflotron Plus Dry Chemistry Analyzer according to the manufacturer’s protocol.

### Hydroxyproline assay

The assessment of Hydroxyproline was performed as described before^45^.

### Potassium-hydroxide digestion

Liver tissue was digested in 5% potassium-hydroxide solution (KOH) at 37 °C. After 16 h, eggs in the solution were counted under the microscope (magnification × 40).

### MDA assay

Measurement of malondialdehyde was performed in liver tissue by the Lipid Peroxidation (MDA) Assay Kit (MAK085) by Sigma-Aldrich according to the manufacturer’s protocol.

### Statistical analysis

Statistical analysis was performed with SPSS 26.0. (SPSS Inc., IBM Corp., Armonk, NY). In regard to the exploratory nature of the current study, one-way ANOVA with post-hoc Fisher's Least Significant Differences (LSD) was used to calculate pairwise differences between animal groups^46^. Absolute values are presented and statistic calculations were performed with transformed data. Data was skewed to the right and thus transformed using the natural logarithm. Relevant *p*-values are indicated in the corresponding figures.

### Supplementary Information

Supplementary Figures.

## Data Availability

The datasets used and/or analyzed during the current study are available from the corresponding author on reasonable request.
