# Supplementary material for: Hepatocyte integrity depends on c-Jun-controlled proliferation in Schistosoma mansoni infected mice
Source: Sci Rep. 2023 Nov 21;13:20390. doi: 10.1038/s41598-023-47646-z (PMC10663609; doi:10.1038/s41598-023-47646-z)

Hepatocyte integrity depends on c-Jun-controlled proliferation in *S. mansoni* infected mice

Lukas Härle[1#], Verena von Bülow[1#], Lukas Knedla[1], Frederik Stettler[1], Heike Müller[1] Daniel Zahner[2], Simone Haeberlein[3], Anita Windhorst[4], Annette Tschuschner[1], Monika Burg-Roderfeld[5], Kernt Köhler[6], Christoph G. Grevelding[3], Elke Roeb[1#], Martin Roderfeld[1#*]

[1]Department of Gastroenterology, Justus Liebig University Giessen, 35392 Giessen, Germany

[2]Central Laboratory Animal Facility, Justus Liebig University Giessen, 81, 35392 Giessen, Germany

[3]Institute of Parasitology, BFS, Justus Liebig University Giessen, 35392 Giessen, Germany

[4]Institute of Medical Informatics, Justus Liebig University Giessen, Rudolf-Buchheim-Str. 6. 35392 Giessen, Germany

[5]Hochschulen Fresenius GmbH, University of Applied Sciences, 65510 Idstein, Germany

[6]Institute of Veterinary Pathology, Justus Liebig University Giessen, Giessen, Germany

# These authors contributed equally.

*Corresponding author: Martin Roderfeld, ORCID 0000-0002-3232-1277, Department of Gastroenterology, Justus Liebig University Giessen, Gaffkystr. 11, 35392 Giessen, Germany. Phone: +49 641 99 42527; Fax: +49 641 99 42333; E-mail: martin.roderfeld@innere.med.uni-giessen.de

Suppl. Figure 1

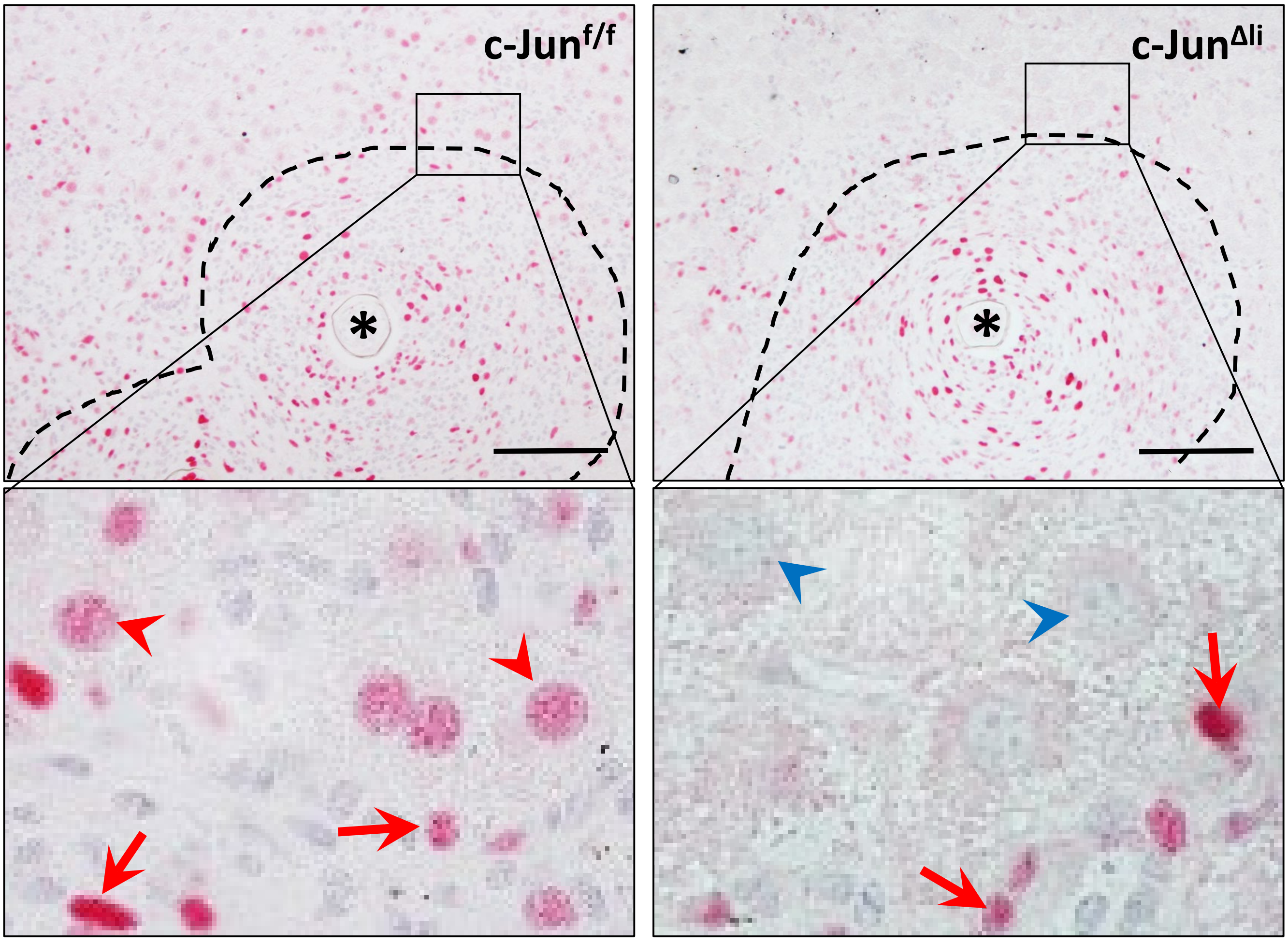

SFig. 1 Enlarged c-Jun immunostaining from Figure 1B.

Suppl. Figure 2

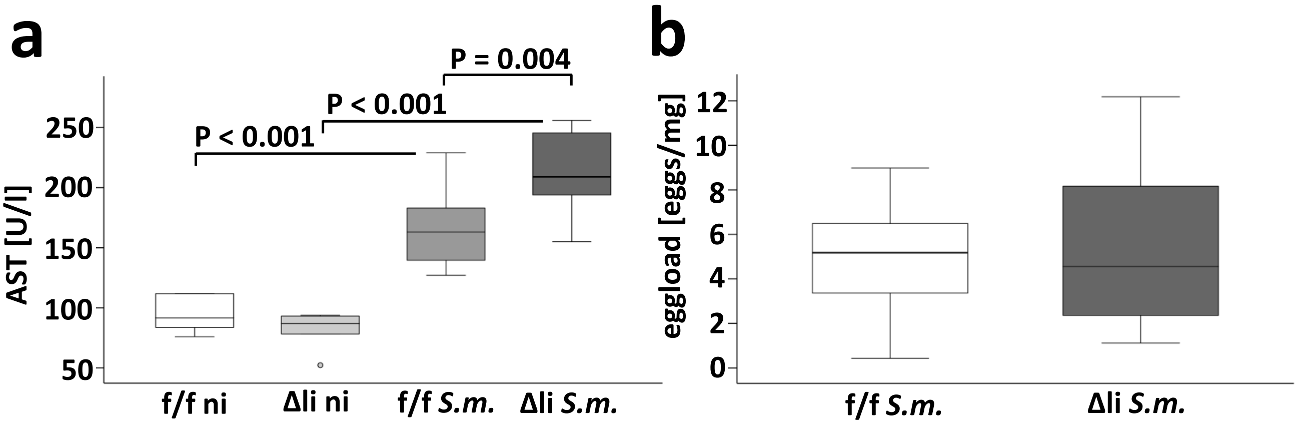

SFig. 2 (A) Serum aspartate transaminases (AST) displayed an elevation from non-infected animals to infected animals and a difference between infected animals. (B) Potassium hydroxide digestion depicted the same *S. mansoni* egg load in infected animals independent of genotype. n=11 f/f *S.m.*, n=12 Δli *S.m*. The indicated p values were calculated by ANOVA and post hoc pairwise comparison of groups using Fisher's LSD on log transformed data.

Suppl. Figure 3

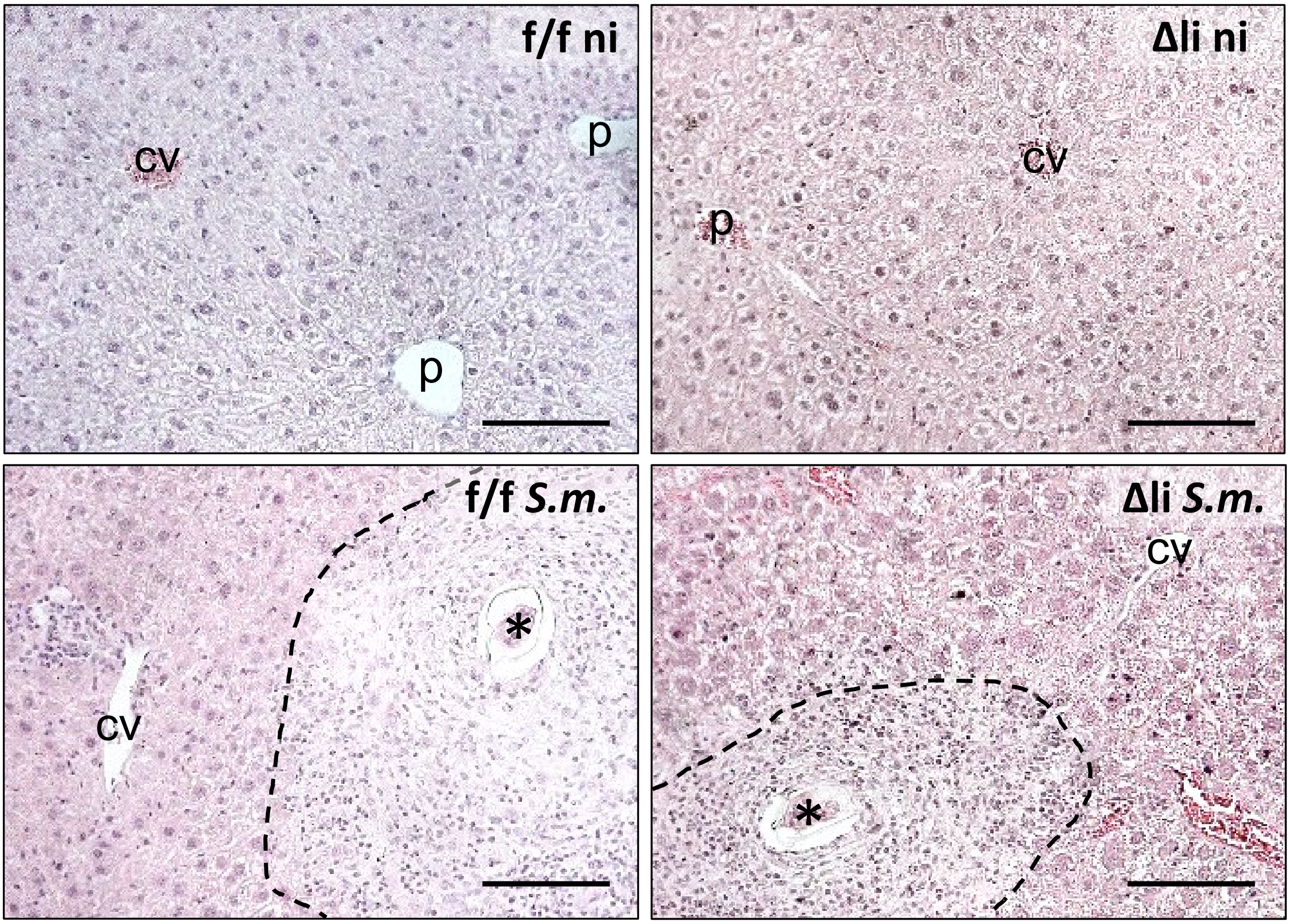

SFig. 3 Enlarged microphotographs of the H&E staining depicted in Figure 2C.

Suppl. Figure 4

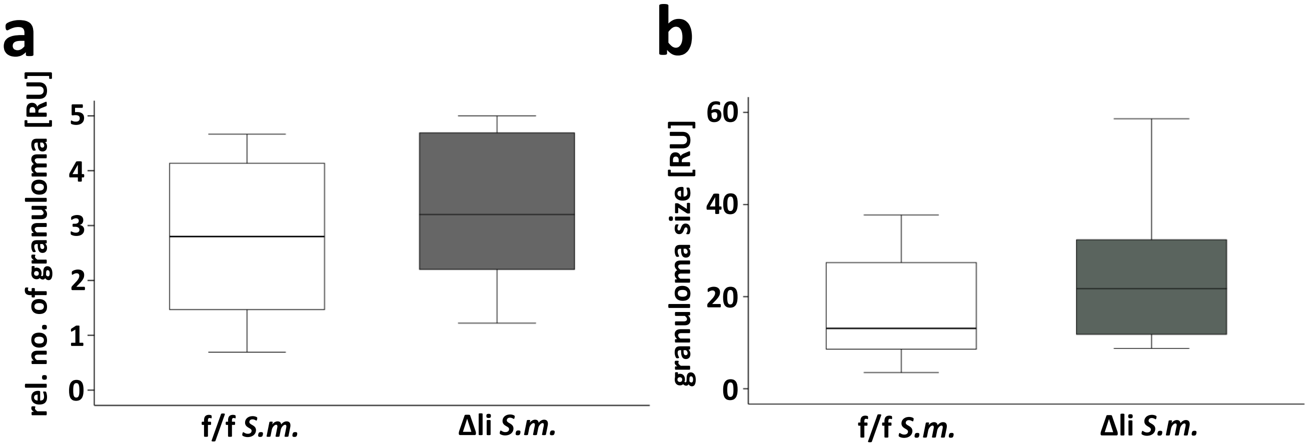

SFig. 4 (A) Histologic assessment of the numbers of hepatic granuloma showed no difference in infected animals independent of the hepatocellular knockout of c-Jun. (B) Morphometric assessment of granuloma size revealed equal dimensions of granuloma in f/f *S.m*. and Δli *S.m*. mice. n=11 f/f *S.m*., n=12 Δli *S.m*.

Suppl. Figure 5

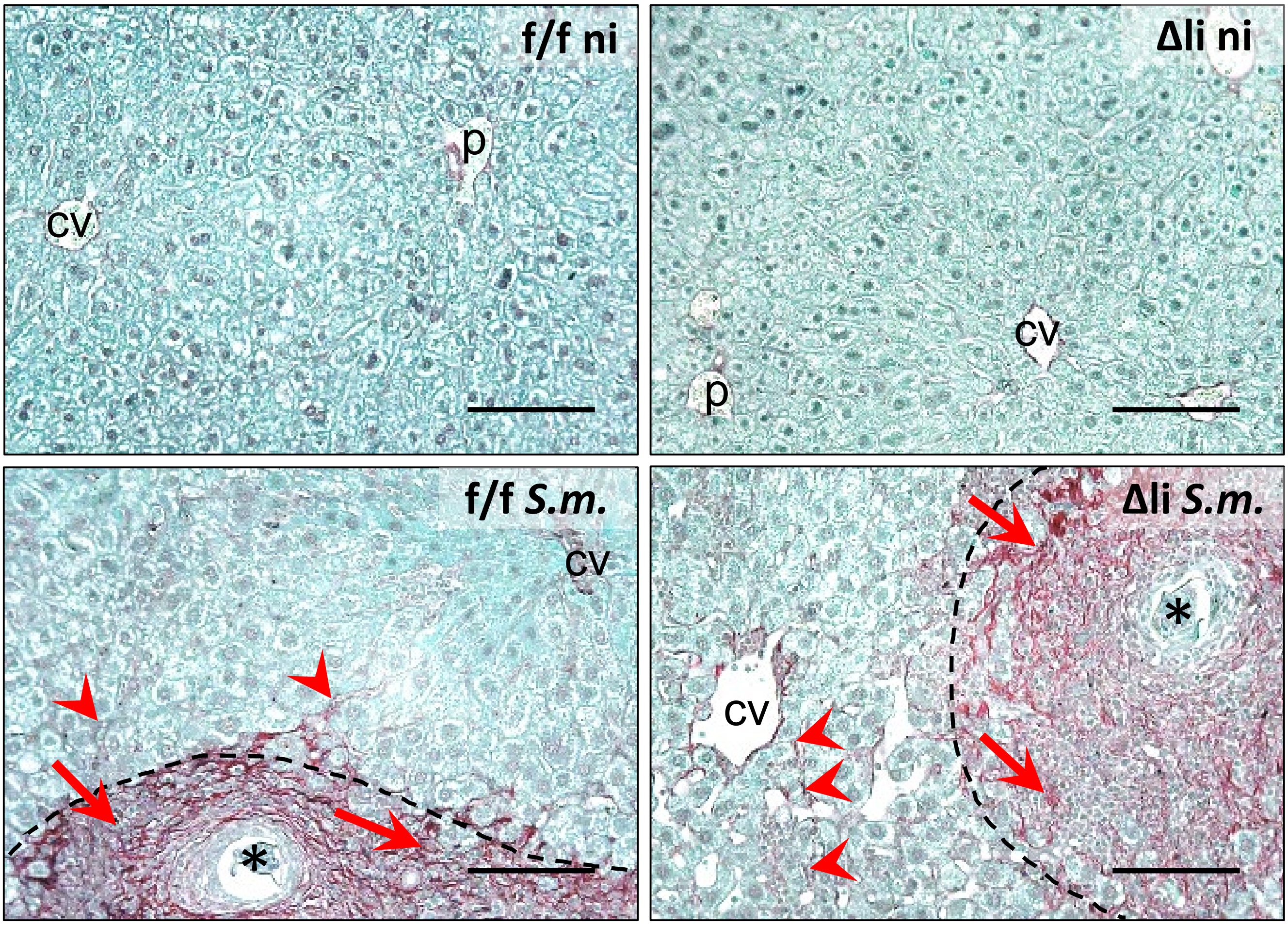

SFig. 5 Enlarged microphotographs of the sirius red stainings shown in Figure 2D.

Suppl. Figure 6

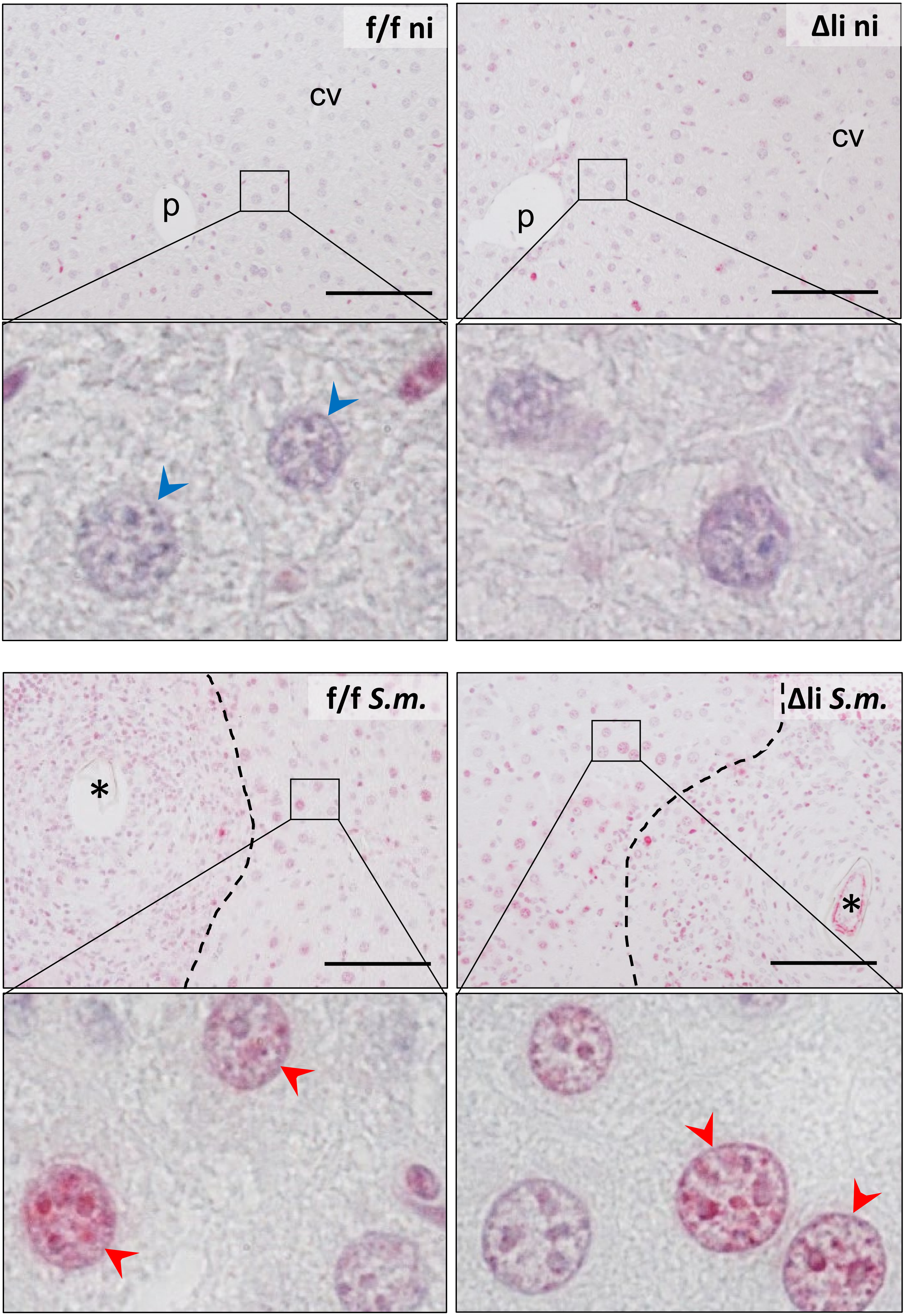

SFig. 6 Enlarged microphotographs of the γ-H2a.X immunostainings demonstrated in Figure 2G.

Suppl. Figure 7

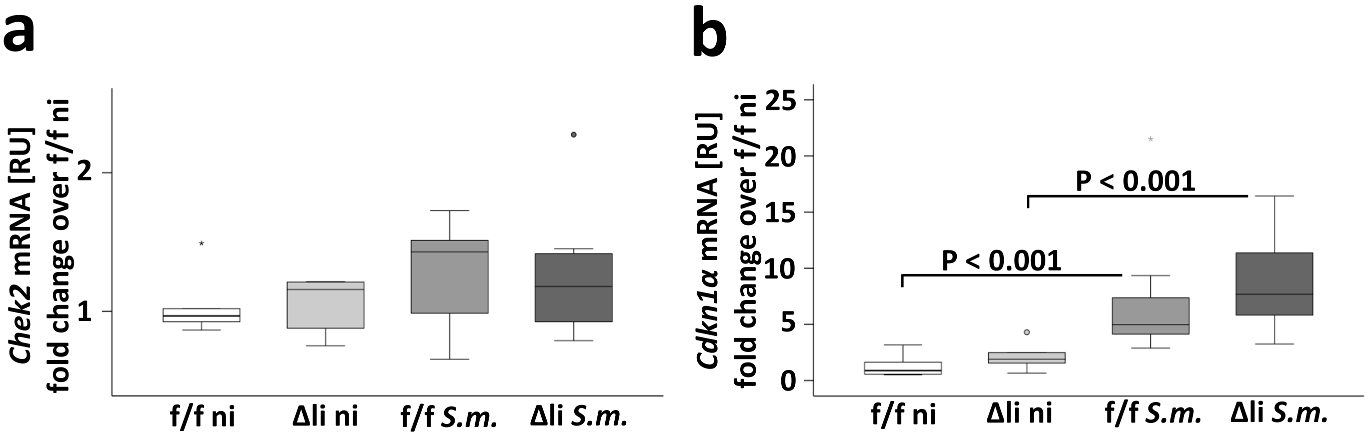

SFig. 7 qRT-PCR validation of qPCR array targets with x-fold values. (A) qRT-PCR results show no change in *Chek2* expression. (B) qRT-PCR results from *Cdkn1a* showed an upregulation upon infection in comparison to non-infected animals, but no difference between infected animals. n=6 f/f ni, n=6 Δli ni, n=11 f/f *S.m.*, n=12 Δli *S.m.*, and 3 technical replications. The indicated p values were calculated by ANOVA and post hoc pairwise comparison of groups using Fisher's LSD on log transformed data.

Suppl. Figure 8

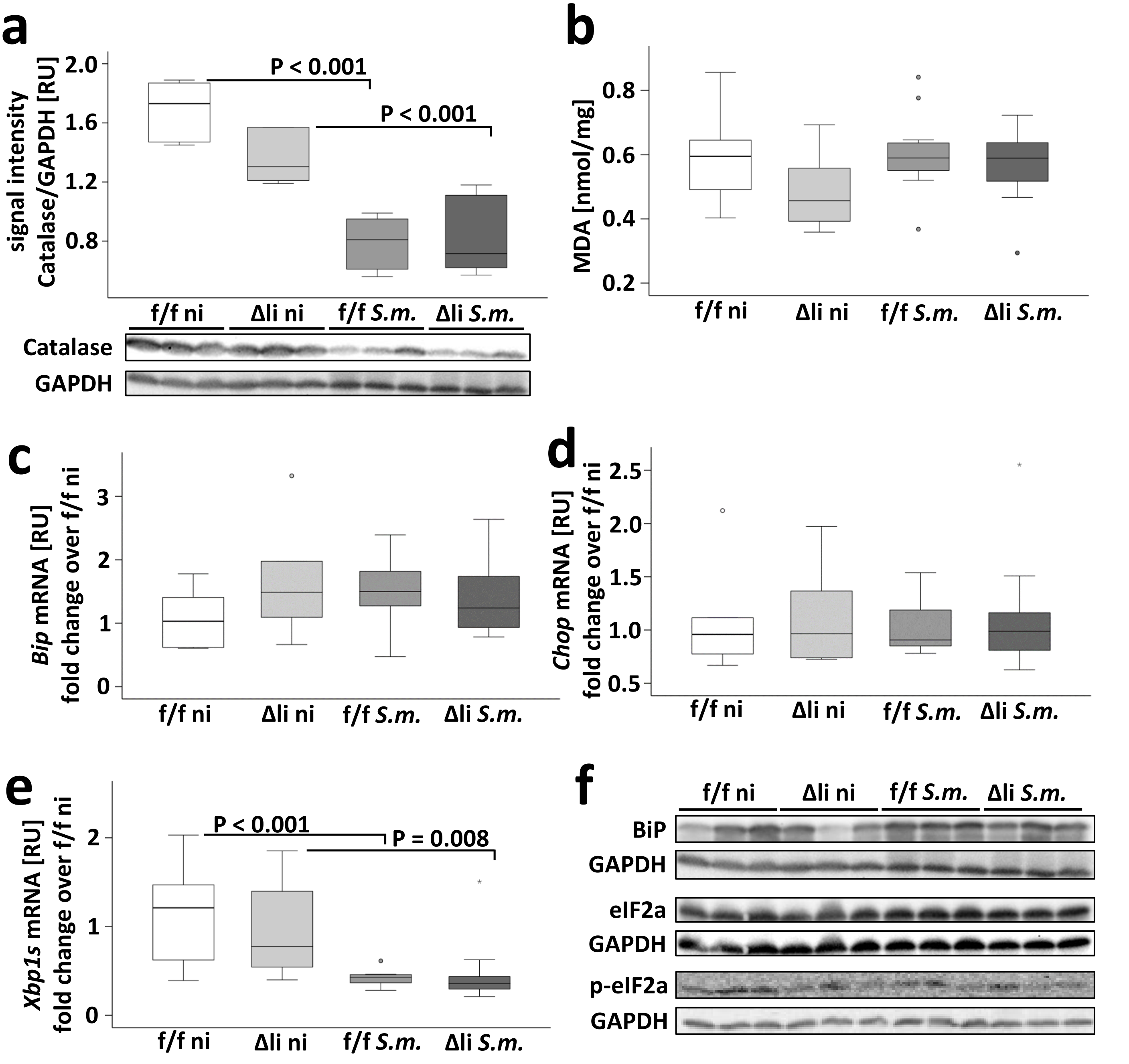

SFig. 8 Regulation of markers for oxidative stress and ER stress. (A) Western blot analysis and subsequent assessment of optical density of the signals depicted lowered expression of the oxidative stress marker catalase in infected animals compared to non-infected animals, but no differences between infected animals. n=6, 3 technical replicates (B) Hepatic malondialdehyde was not altered. (C, D) ER stress markers *Bip* and *Chop* were equally regulated in all groups. (E) Hepatic expression of the ER stress marker *Xbp1s* was reduced upon infection with *S. mansoni*. (C-E) n=6 f/f ni, n=6 Δli ni, n=11 f/f *S.m.*, n=12 Δli *S.m* and 3 technical replications (F) ER stress markers BiP, eIF2a and p-eIF2a showed no alterations in expression over all animal groups. n=6, 3 technical replicates. The indicated p values were calculated by ANOVA and post hoc pairwise comparison of groups using Fisher's LSD on log transformed data.

Suppl. Figure 9

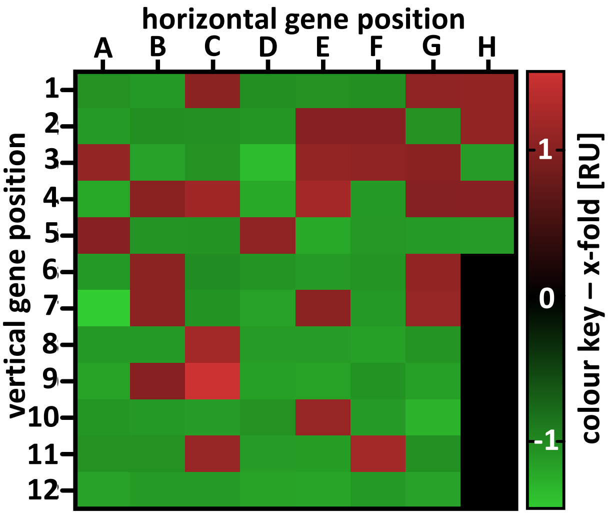

horizontal gene position

vertical gene position

Layout	A	B	C	D	E	F	G	H
1	Akt1 0.96	Atg5 0.92	Casp3 1.05	Eif2ak3 0.98	Hsp90aa1 0.96	Mtor 0.98	Snca 1.07	Actb 1.09
2	Ambra1 0.91	Atg7 0.98	Casp8 0.97	Eif4g1 0.93	Hspa8 1.01	Nfkb1 1.02	Sqstm1 0.97	B2m 1.09
3	App 1.11	Atg9a 0.87	Cdkn1b 0.96	Esr1 0.74	Htt 1.11	Npc1 1.07	Tgfb1 1.03	Gapdh 0.9
4	Atg10 0.84	Atg9b 1.03	Cdkn2a 1.19	Fadd 0.83	Ifng 1.23	Pik3c3 0.91	Tgm2 1.00	Gusb 1.02
5	Atg12 1.01	Bad 0.95	Cln3 0.95	Fas 1.07	Igf1 0.82	Pik3cg 0.93	Tmem74 0.91	Hsp90ab1 0.9
6	Atg16l1 0.91	Bak1 1.05	Ctsb 0.99	Gaa 0.94	Ins2 0.91	Pik3r4 0.94	Tnf 1.09	controll
7	Atg16l2 0.68	Bax 1.05	Ctsd 0.95	Gabarap 0.87	Irgm1 1.05	Prkaa1 0.92	Tnfsf10 1.12	controll
8	Atg3 0.92	Bcl2 0.92	Ctss 1.22	Gabarapl1 0.9	Lamp1 0.9	Pten 0.88	Trp53 0.95	controll
9	Atg4a 0.87	Bcl2l1 1.01	Cxcr4 1.54	Gabarapl2 0.87	Map1lc3a 0.86	Rab24 0.95	Ulk1 0.87	controll
10	Atg4b 0.94	Becn1 0.92	Dapk1 0.89	Hdac1 0.96	Map1lc3b 1.13	Rb1 0.91	Ulk2 0.78	controll
11	Atg4c 0.96	Bid 0.96	Dram1 1.13	Hdac6 0.89	Mapk14 0.88	Rgs19 1.22	Uvrag 0.97	controll
12	Atg4d 0.87	Bnip3 0.91	Dram2 0.91	Hgs 0.86	Mapk8 0.86	Rps6kb1 0.92	Wipi1 0.87	controll

SFig. 9 Markers for autophagy were not regulated in a qPCR array upon c-Jun knockout. (A) 84 genes related to autophagy were analyzed by qPCR array. The signal for each gene was normalized with housekeeping genes and respective x-fold values from infected c-Jun$^{f/f}$ and infected c-Jun$^{\Delta li}$ animals were used to draw the heat map.

Suppl. Figure 10

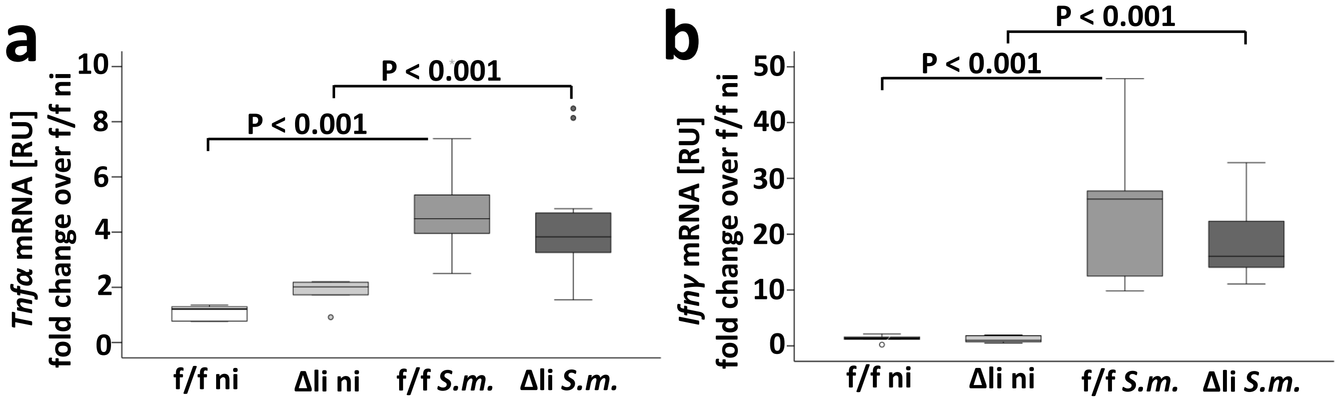

SFig. 10 Markers for hepatic inflammation were induced by *S. mansoni* infection and equally regulated in infected c-Jun$^{f/f}$ and infected c-Jun$^{\Delta li}$ animals. TH1-specific cytokines *Tnf-α* and *Ifn-γ* showed an elevated expression in infected animals, but no difference between the groups of infected animals. n=6 f/f ni, n=6 Δli ni, n=11 f/f *S.m.*, n=12 Δli *S.m.*, 3 technical replicates. The indicated p values were calculated by ANOVA and post hoc pairwise comparison of groups using Fisher's LSD on log transformed data.

Suppl. Figure 11

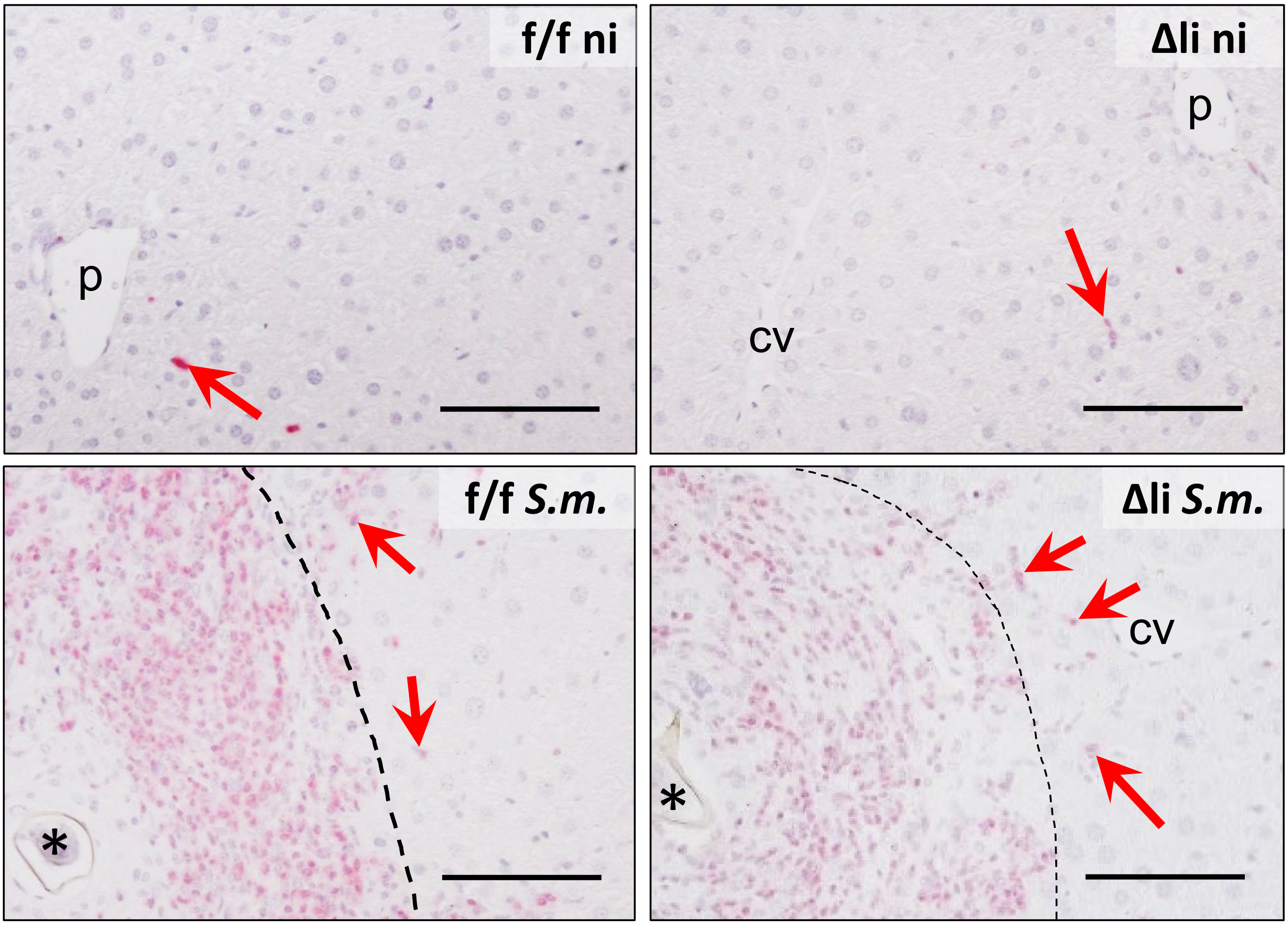

SFig. 11 Enlarged CD45 immunostaining from Figure 4F.

Suppl. Figure 12

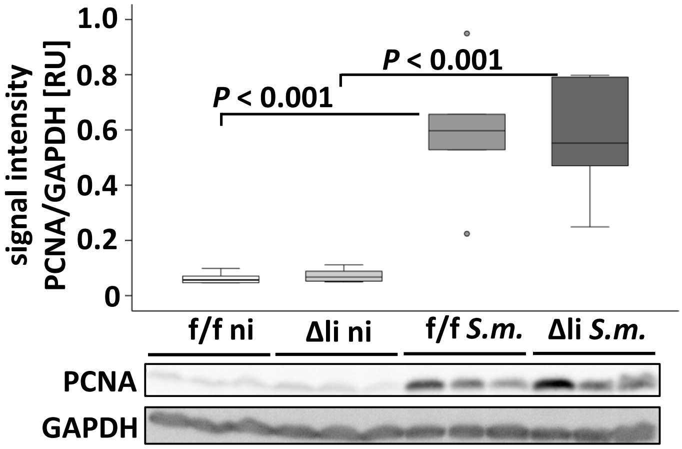

SFig. 12 Western blot analysis and subsequent assessment of optical density of the signals depicted elevated amounts of PCNA, a marker for proliferation, in infected groups compared to the non infected animals. n=6 and 3 technical replicates each. The indicated *P* values were calculated by ANOVA and post hoc pairwise comparison of groups using Fisher's LSD on log transformed data.

Suppl. Figure 13

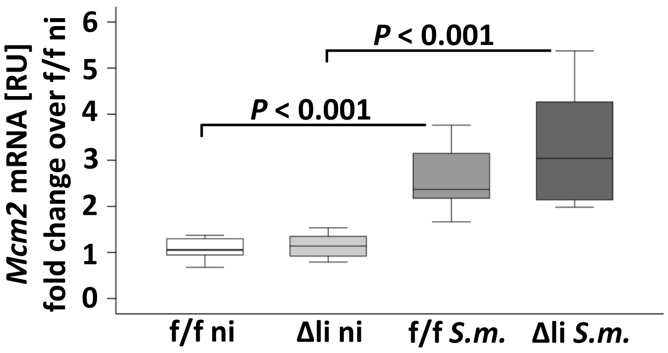

SFig. 13 Mcm2 was induced upon infection in comparison to non infected animals, but no difference between infected animals was observed. n=6 f/f ni, n=6 Δli ni, n=11 f/f *S.m.*, n=12 Δli *S.m.*, 3 technical replicates. The indicated p values were calculated by ANOVA and post hoc pairwise comparison of groups using Fisher's LSD on log transformed data.

Suppl. Figure 14

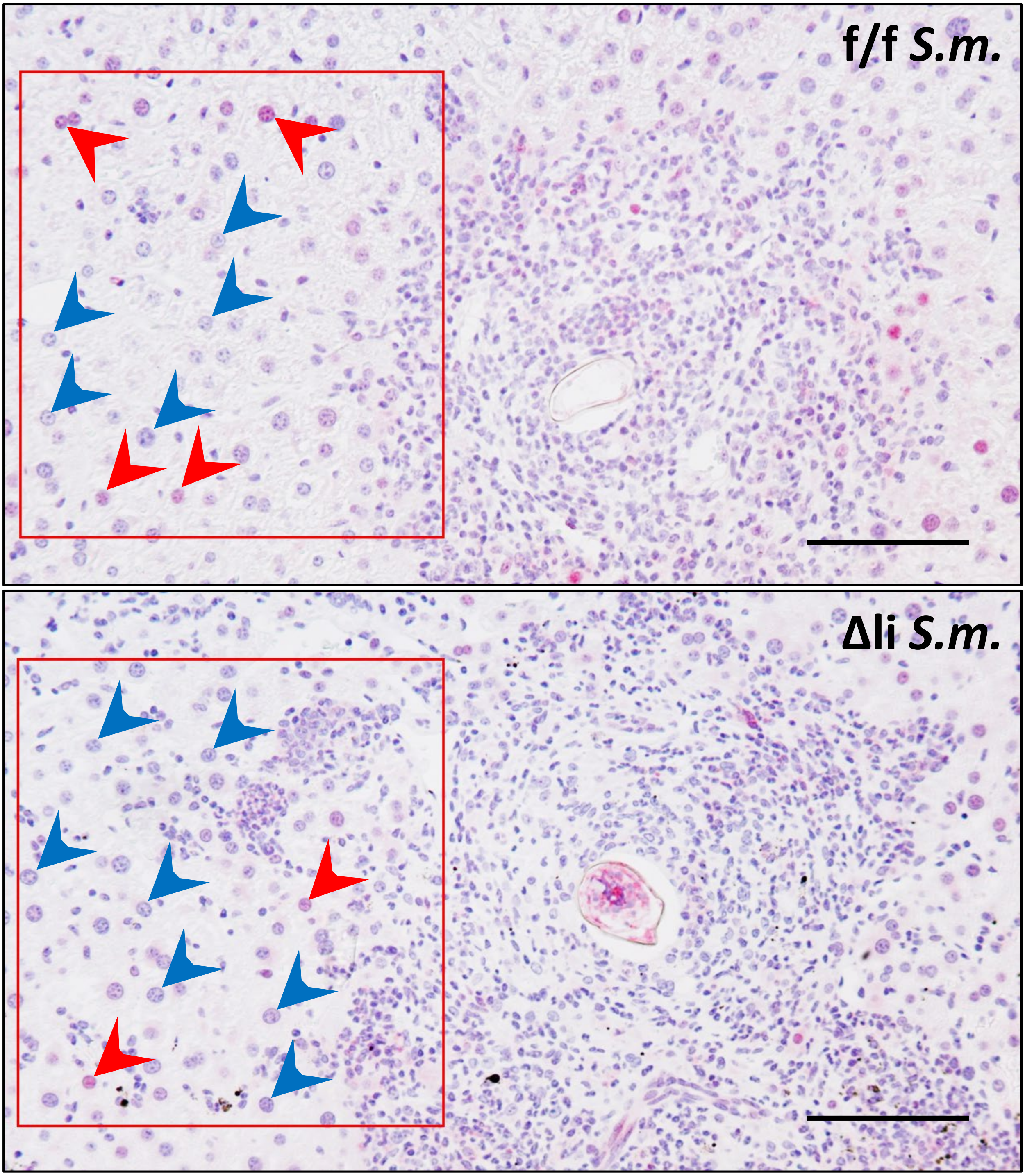

SFig. 14 Representative Cyclin D1 immunostainings – Enlarged pictures of the microphotographs shown in Fig. 5B.

Suppl. Figure 15

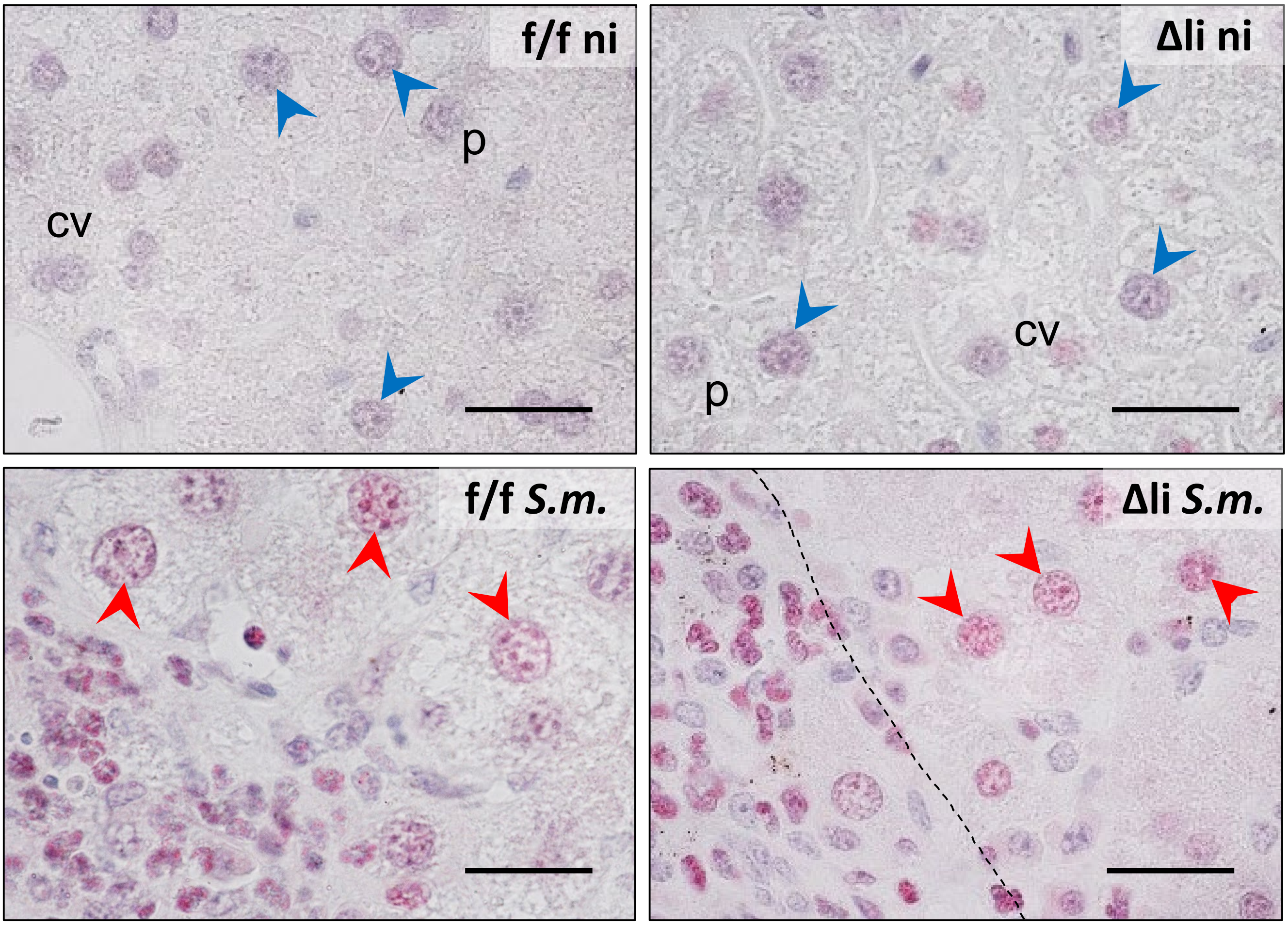

SFig. 15 Enlarged Ki67 immunostaining from Figure 5D.

Suppl. Figure 16

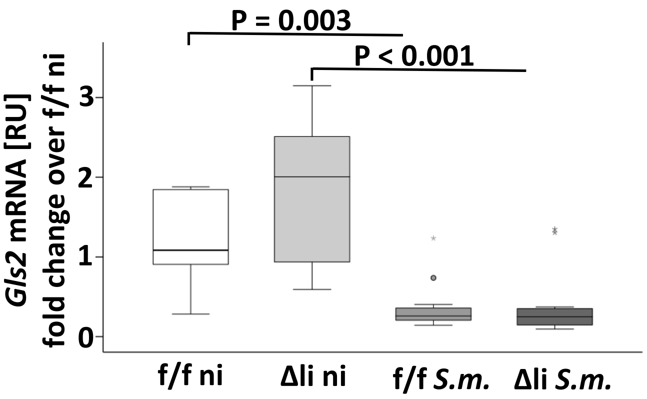

SFig. 16 Glutaminase (*Gls2)* expression is reduced by *S. mansoni* infection and equally regulated in infected c- Jun$^{f/f}$ and infected c-Jun$^{\Delta li}$ animals. qRT-PCR results from *Gls2* showed an reduced expression in infected animals compared to non-infected animals, but no difference between infected animals. n=6 f/f ni, n=6 Δli ni, n=11 f/f *S.m.*, n=12 Δli *S.m.*, 3 technical replicates. The indicated p values were calculated by ANOVA and post hoc pairwise comparison of groups using Fisher's LSD on log transformed data.

Original Blots:

Fig 1C c-Jun (Intas ECL Chemostar) GAPDH (Intas ECL Chemostar)

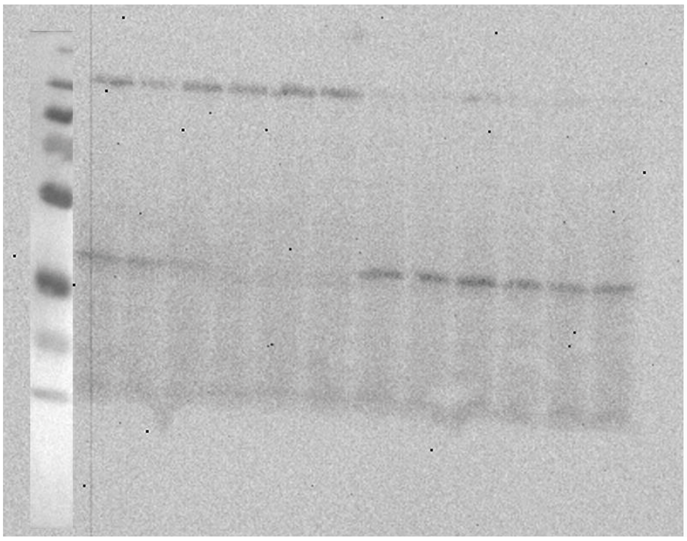

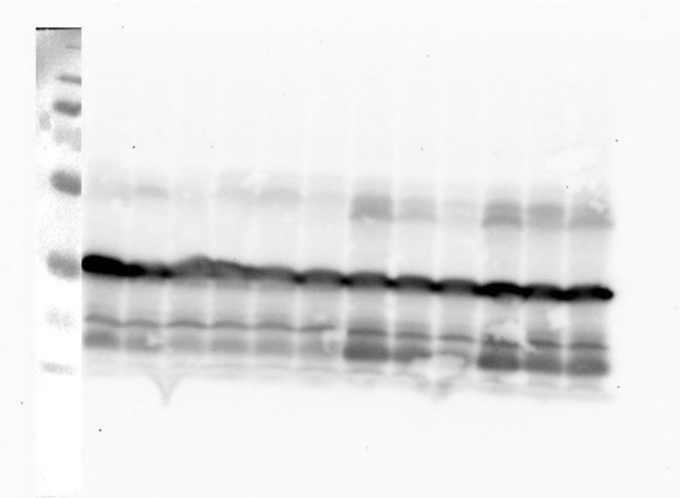

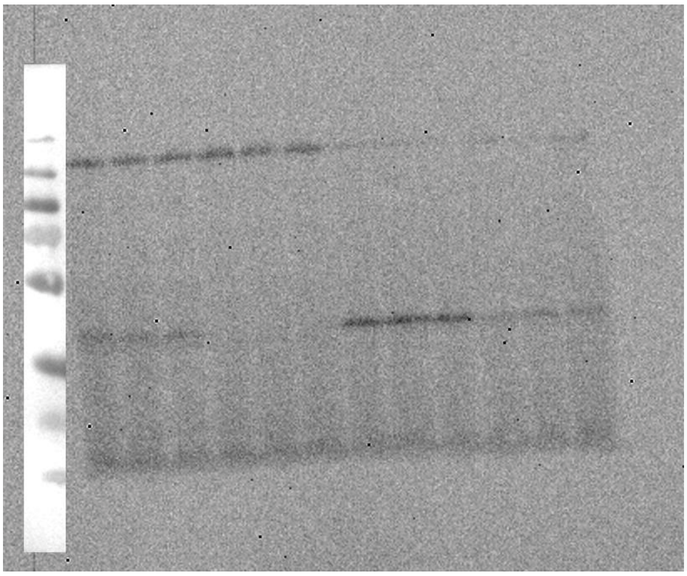

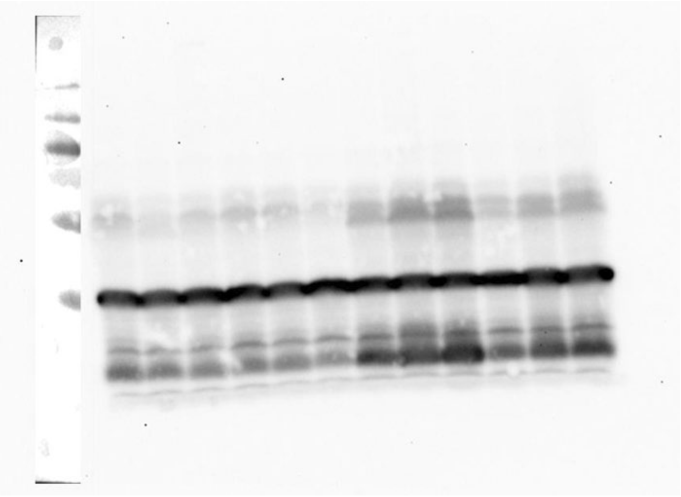

Repetitions

Fig 1C c-Jun (Intas ECL Chemostar) GAPDH (Intas ECL Chemostar)

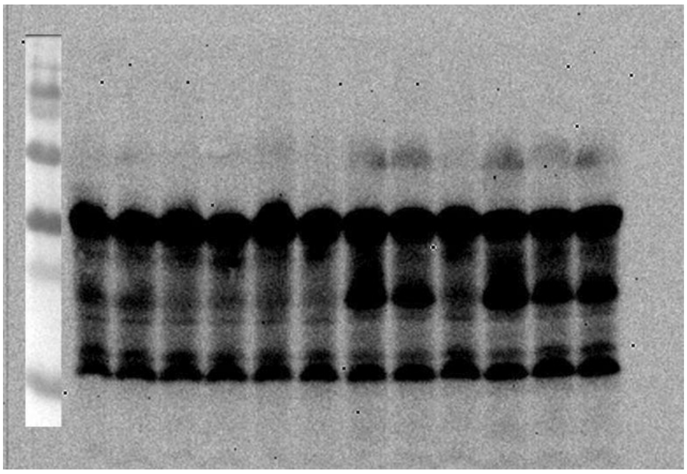

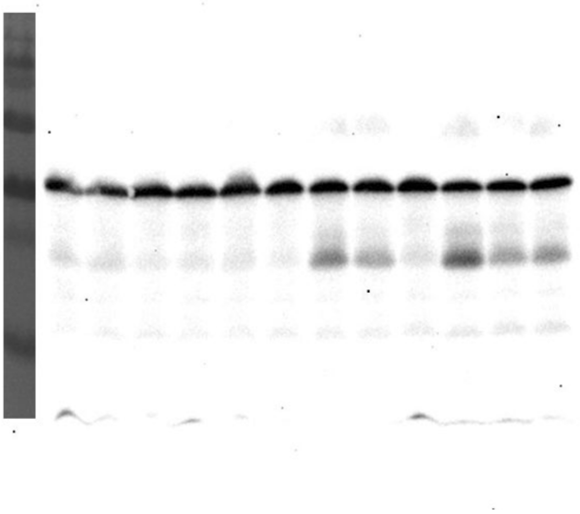

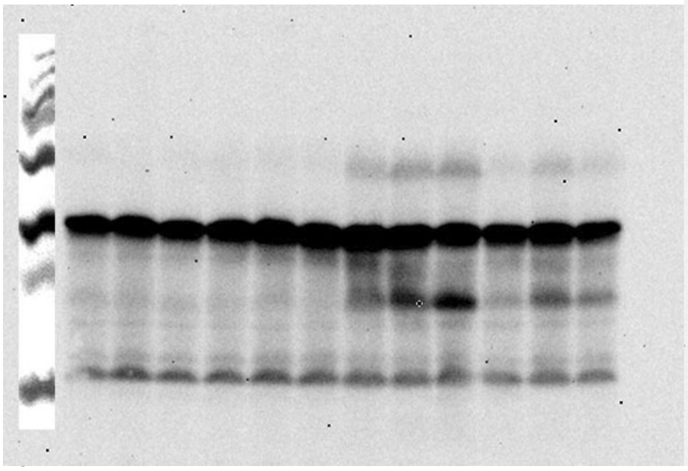

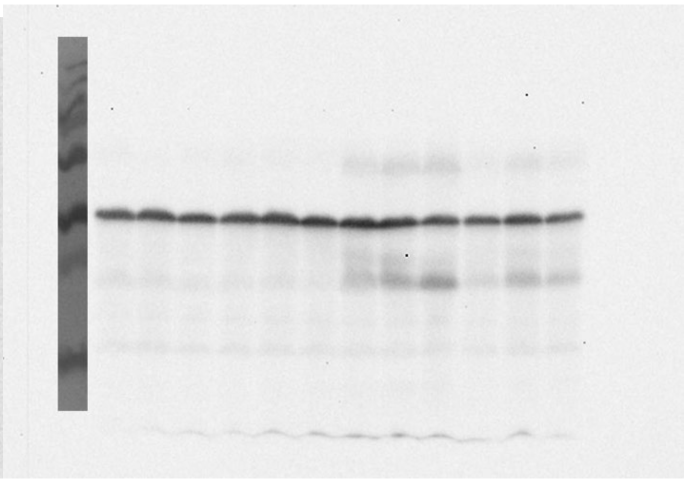

Repetitions

Fig 1C c-Jun (Intas ECL Chemostar)

Alpha-Tubulin (Intas ECL Chemostar)

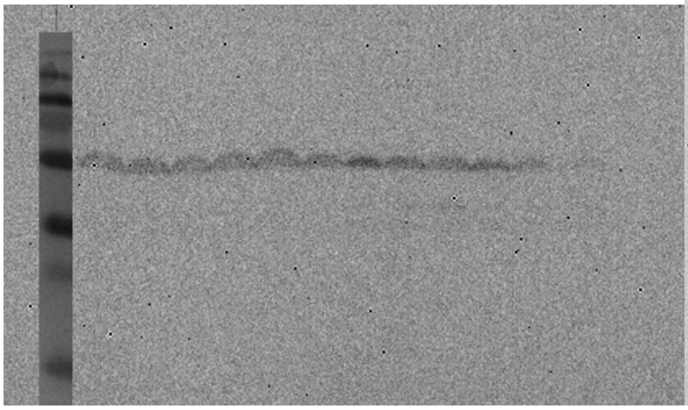

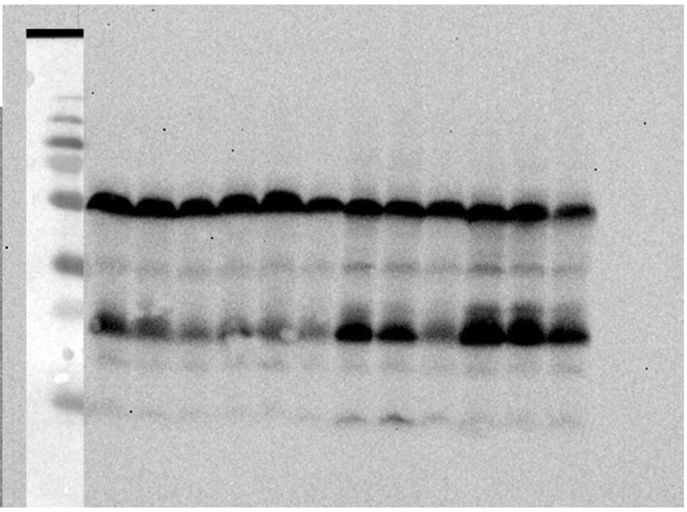

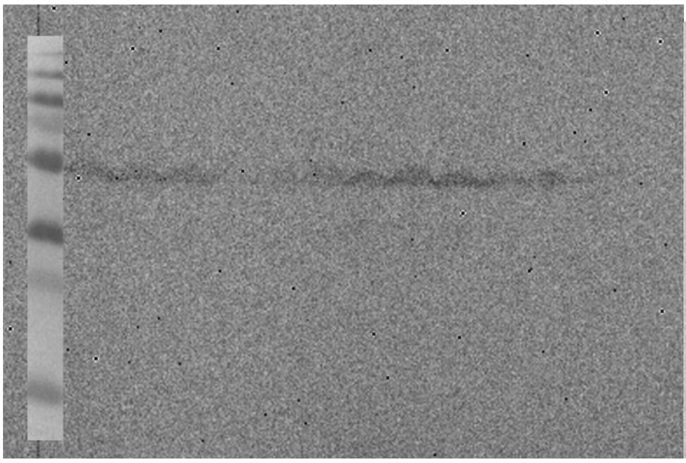

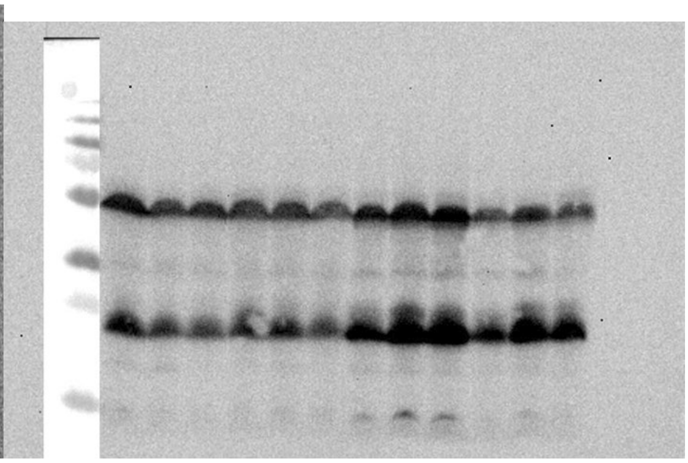

Original Blots

Fig 2F γ-H2a.X (Intas ECL Chemostar)

GAPDH (Intas ECL Chemostar)

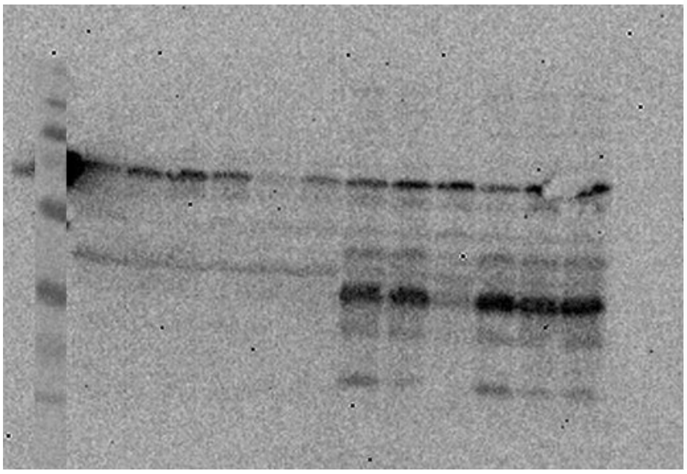

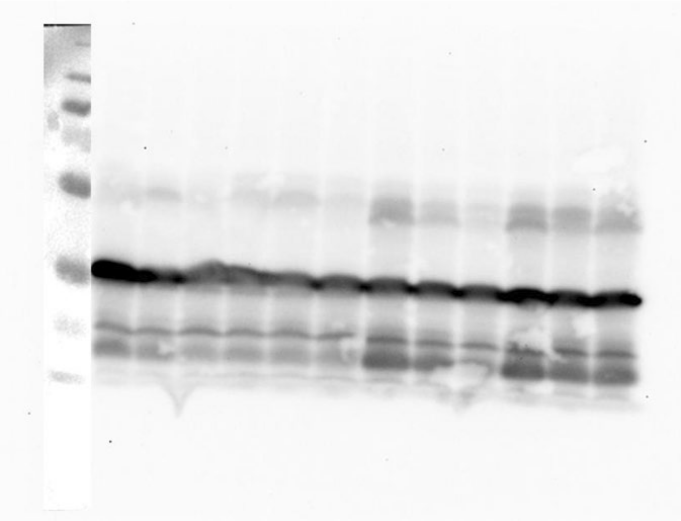

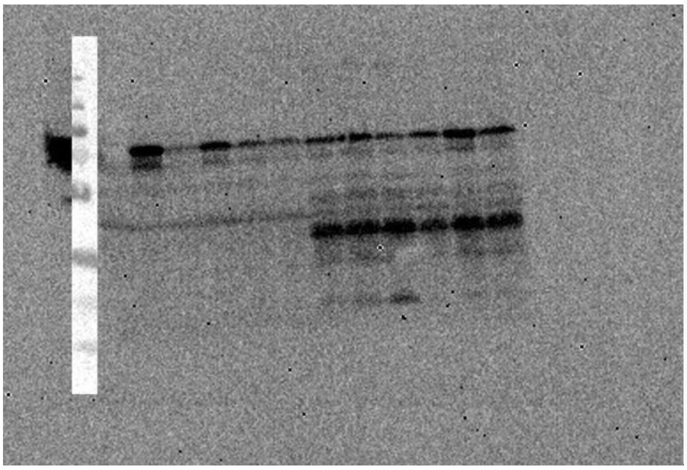

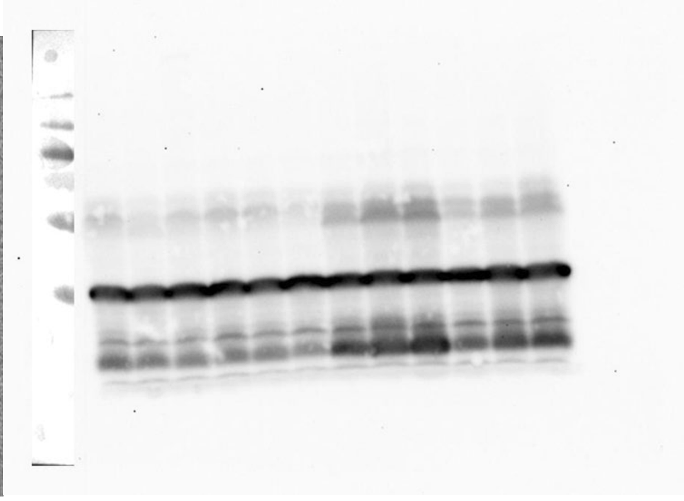

Repetitions

Fig 2F γ-H2a.X (Intas ECL Chemostar)

GAPDH (Intas ECL Chemostar)

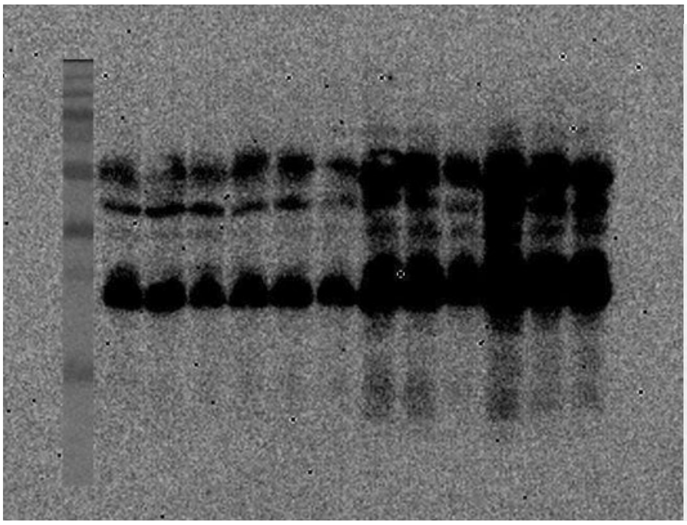

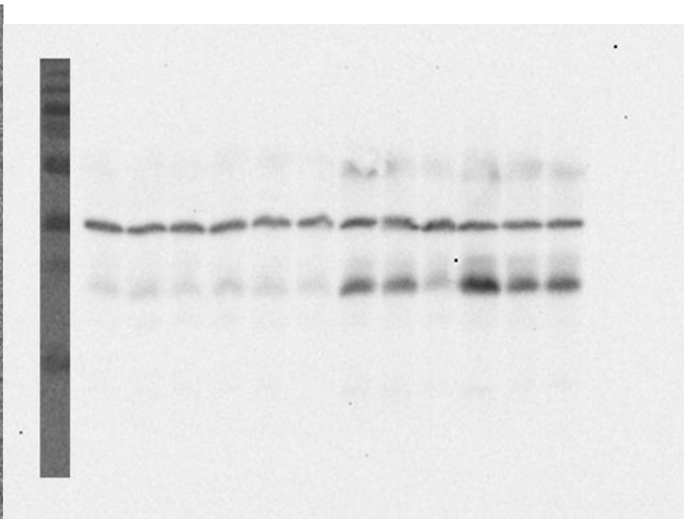

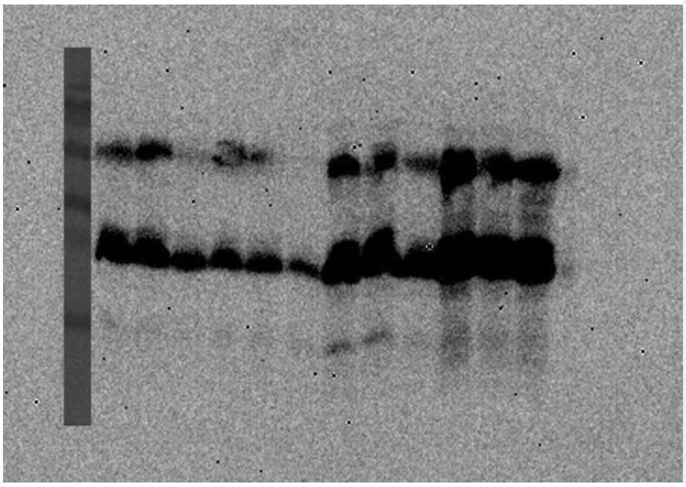

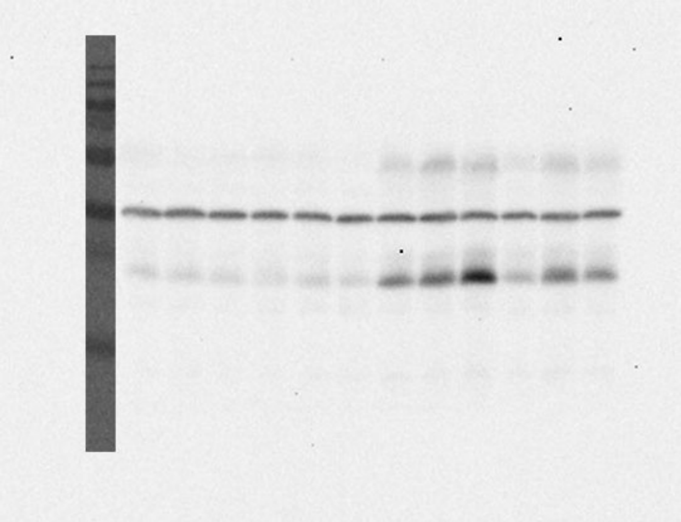

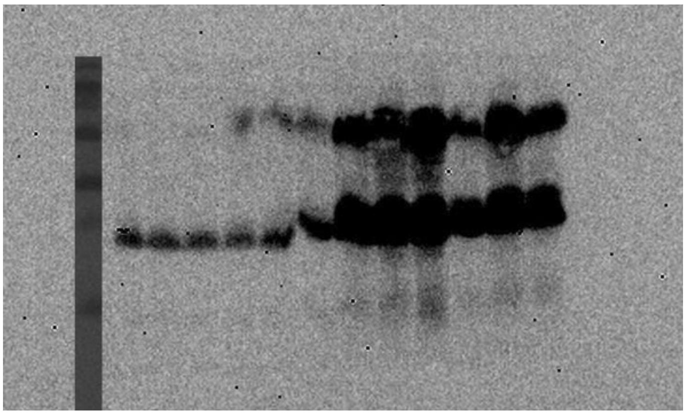

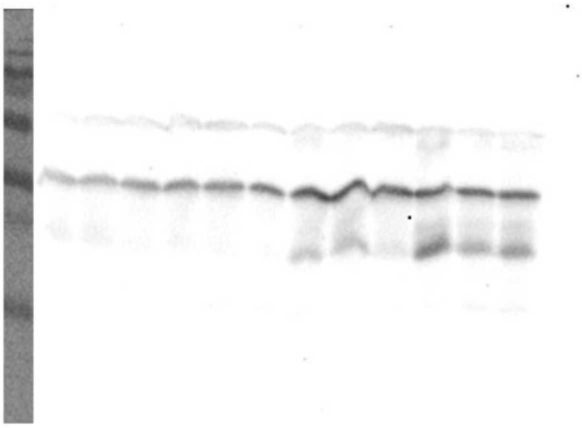

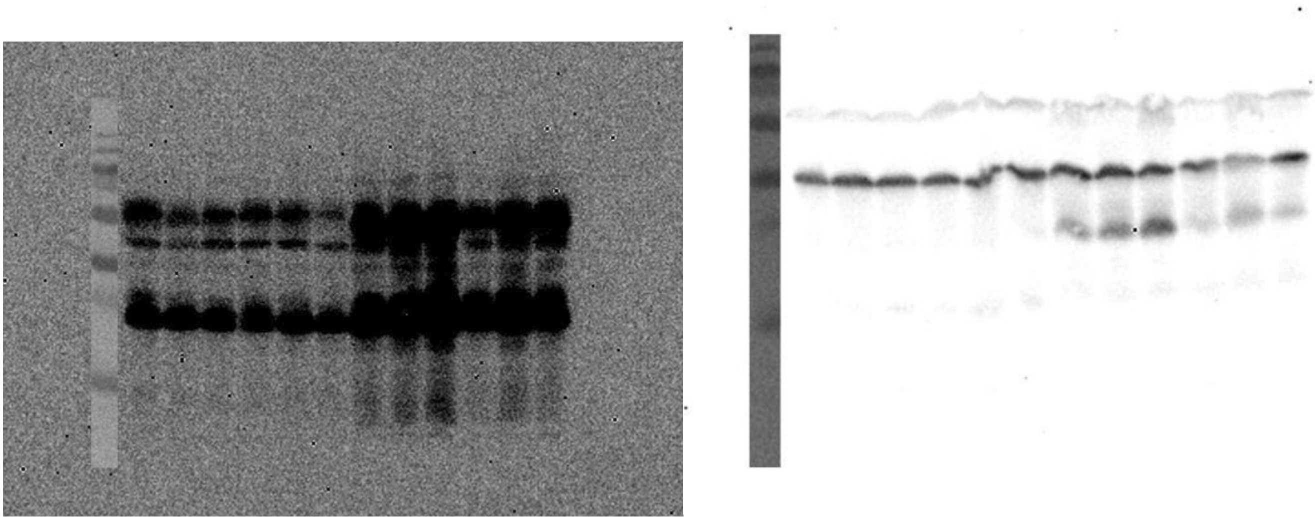

Original Blots

Fig 4E CD45 (Intas ECL Chemostar) GAPDH (Intas ECL Chemostar)

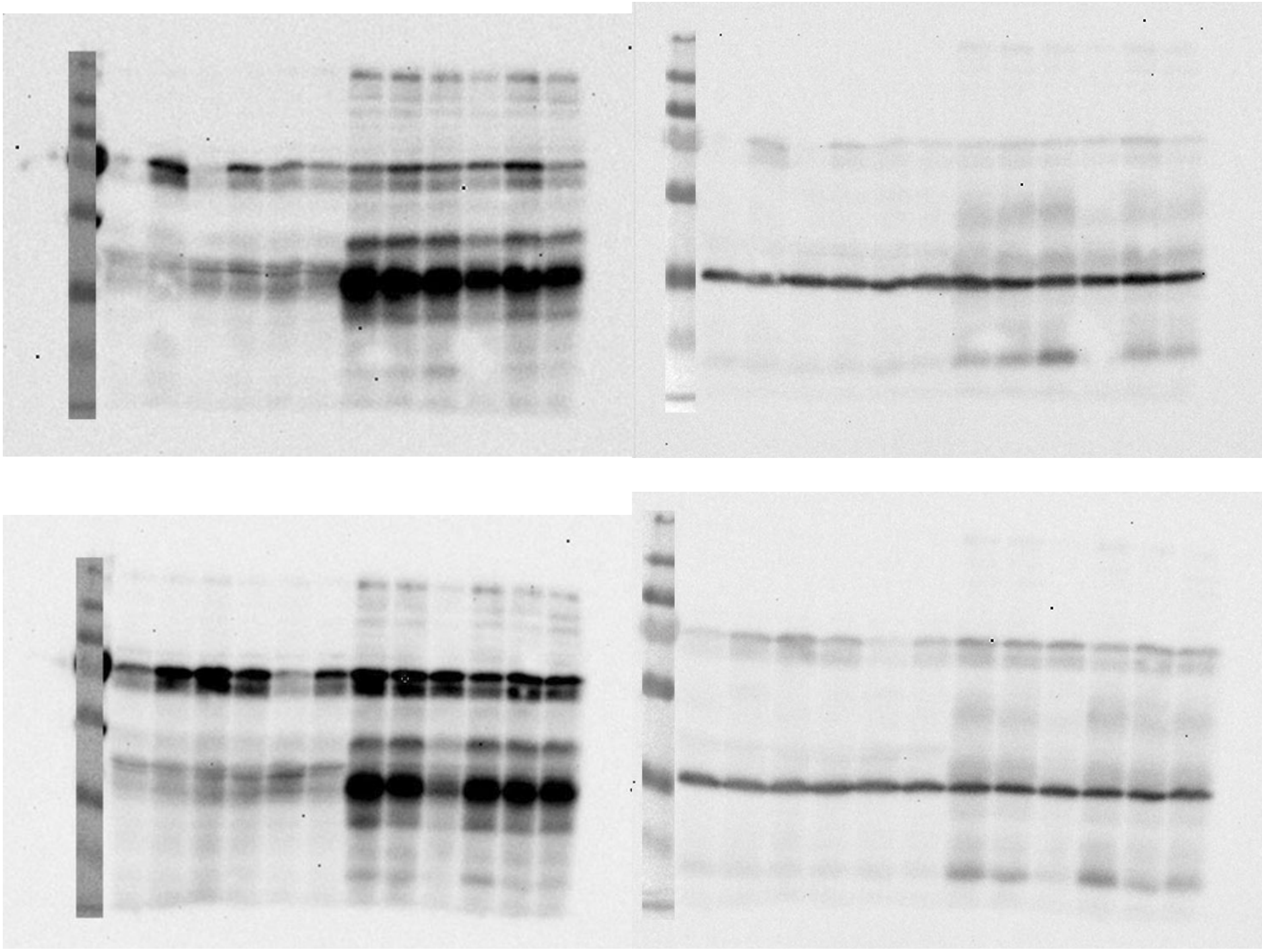

Repetitions

Fig 4E CD45 (Intas ECL Chemostar) GAPDH (Intas ECL Chemostar)

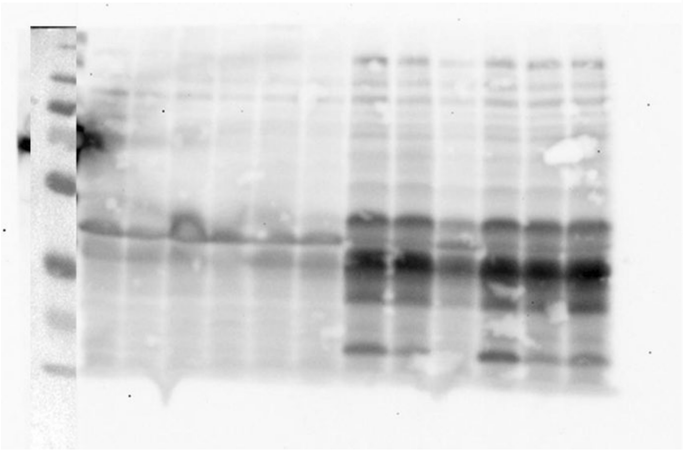

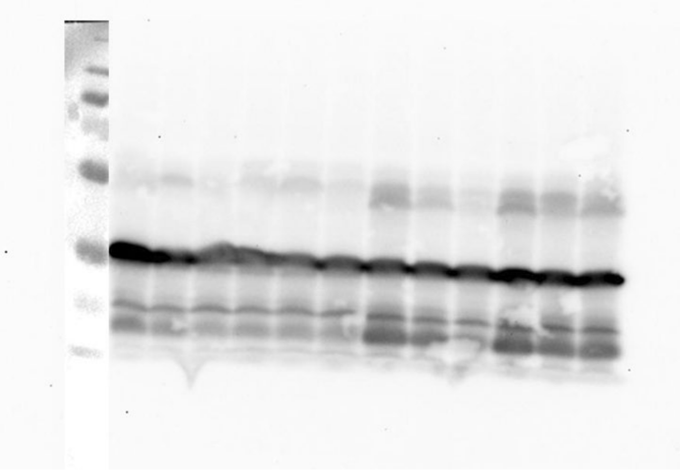

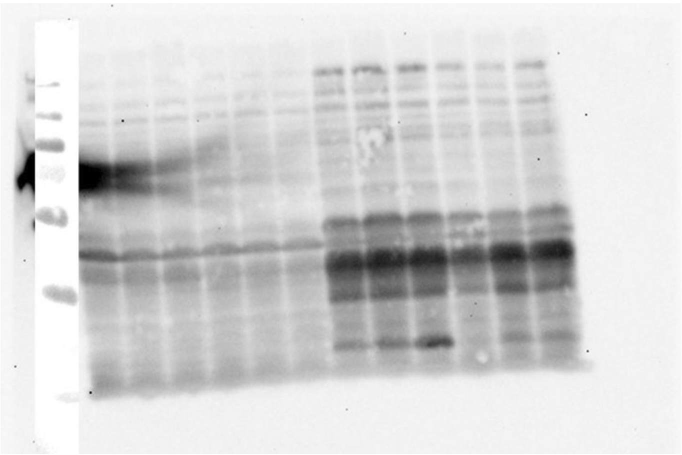

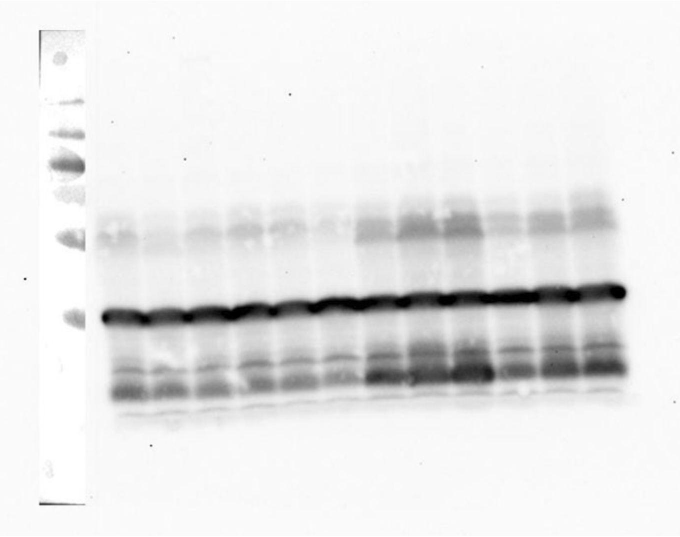

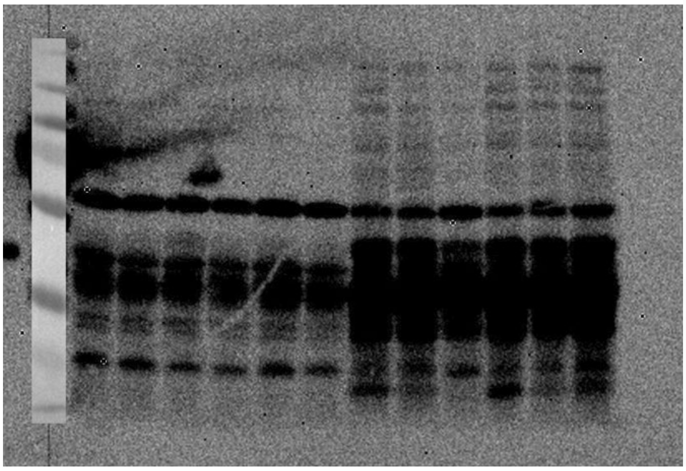

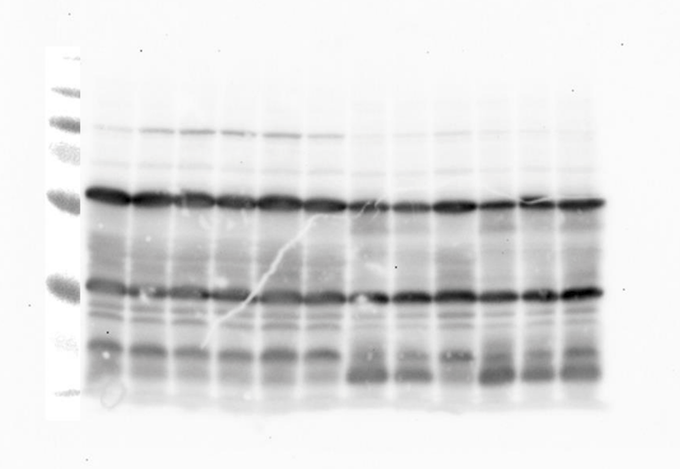

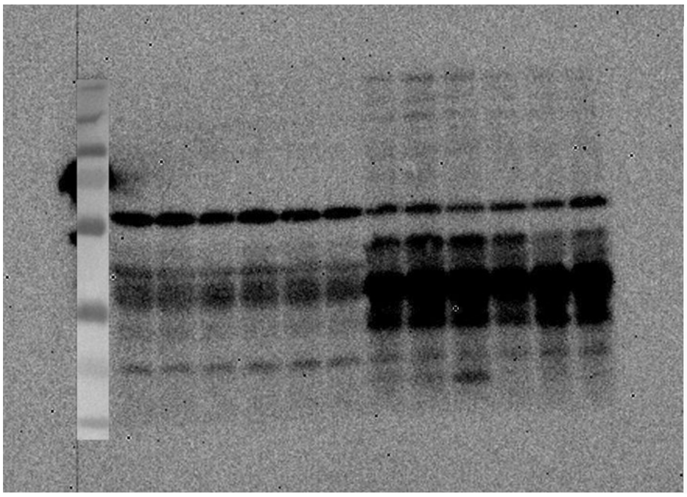

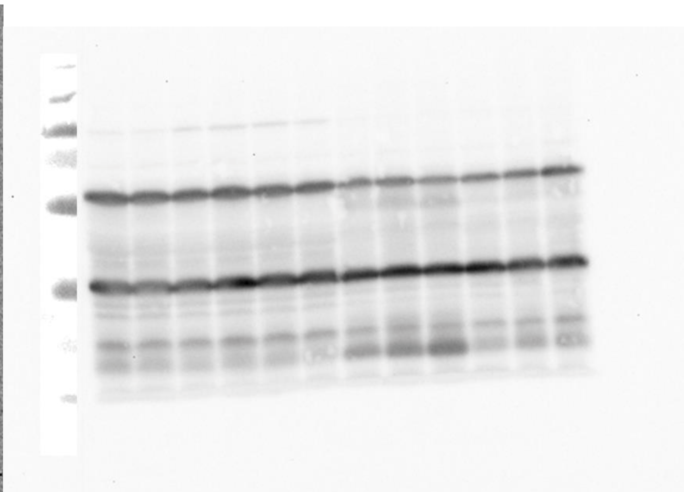

Original Blots

Fig. 5c Cyclin D1 (*X-Ray*)

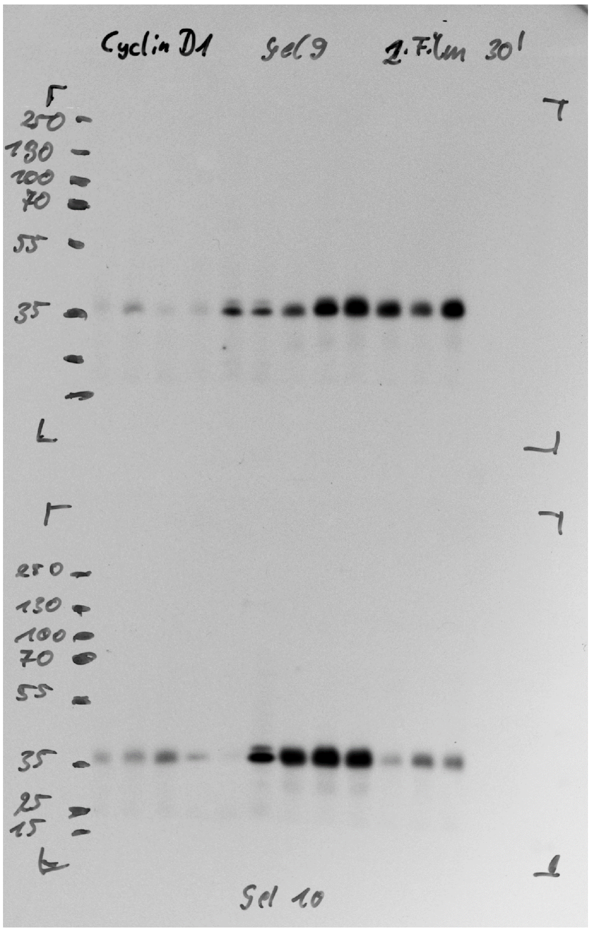

GAPDH (*Intas ECL Chemostar*)

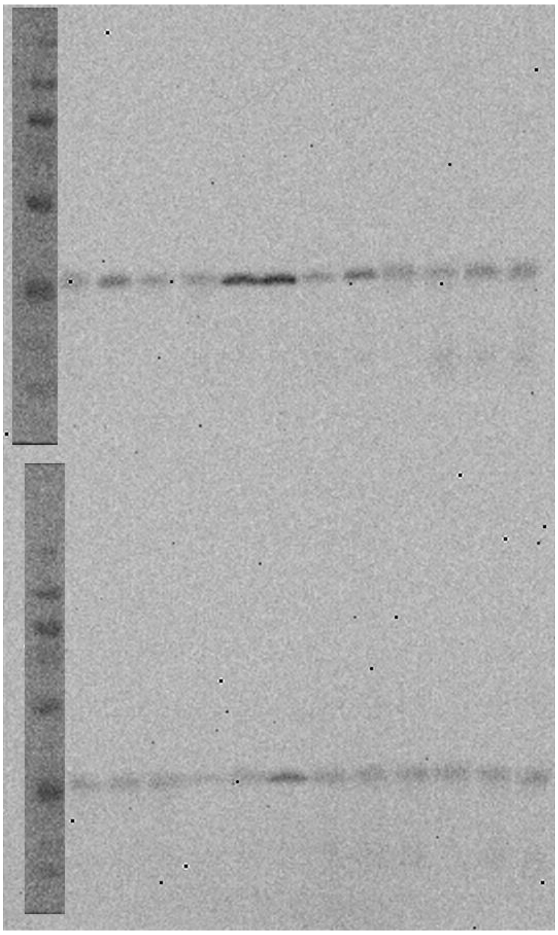

Repetitions

Cyclin D1 (*X-Ray*)

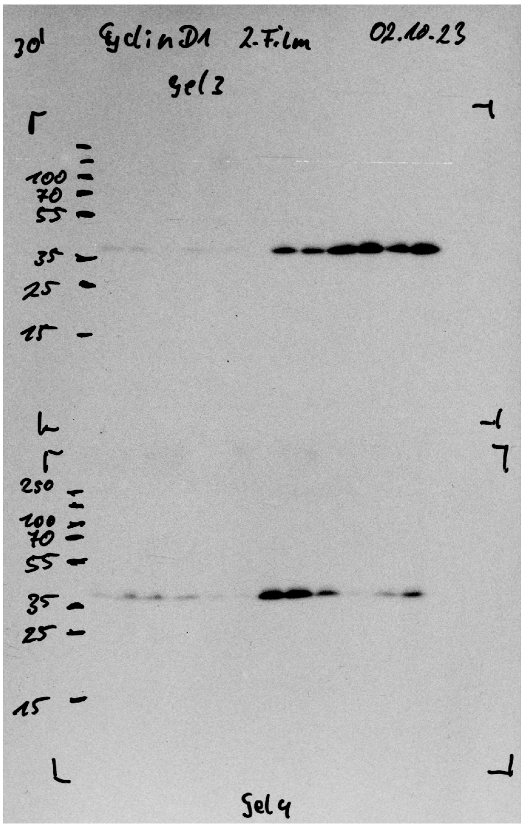

GAPDH (*Intas ECL Chemostar*)

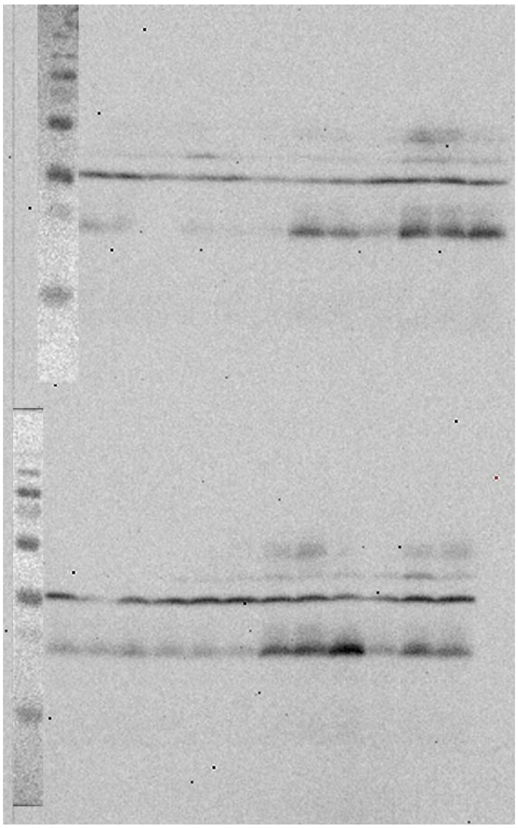

Cyclin D1 (*Intas ECL Chemostar*)

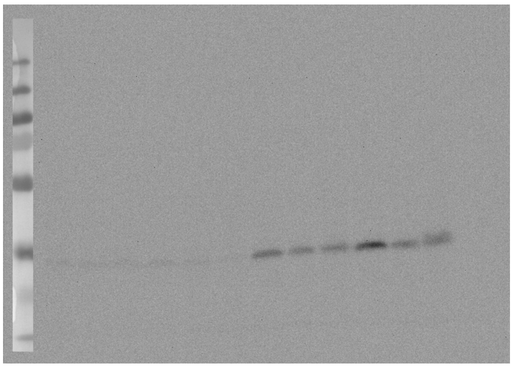

GAPDH (*Intas ECL Chemostar*)

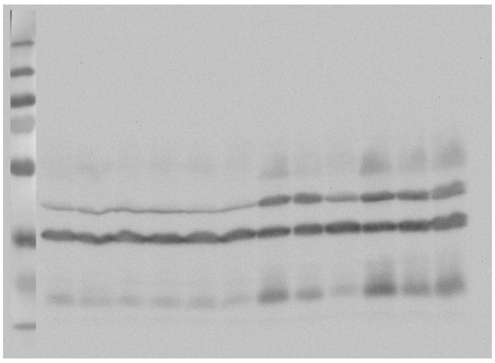

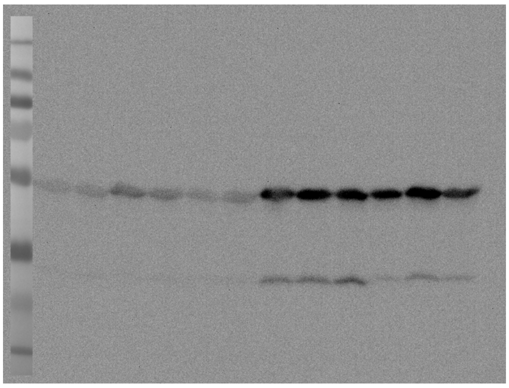

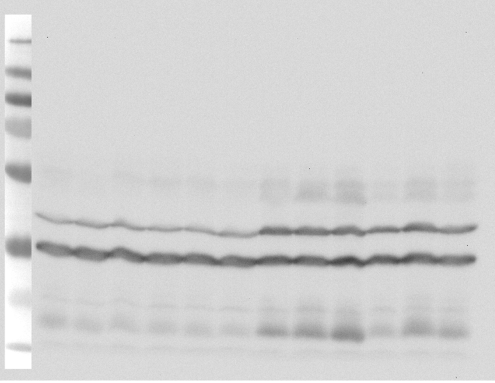

Cyclin D1 (*X-Ray*)

GAPDH (*Intas ECL Chemostar*)

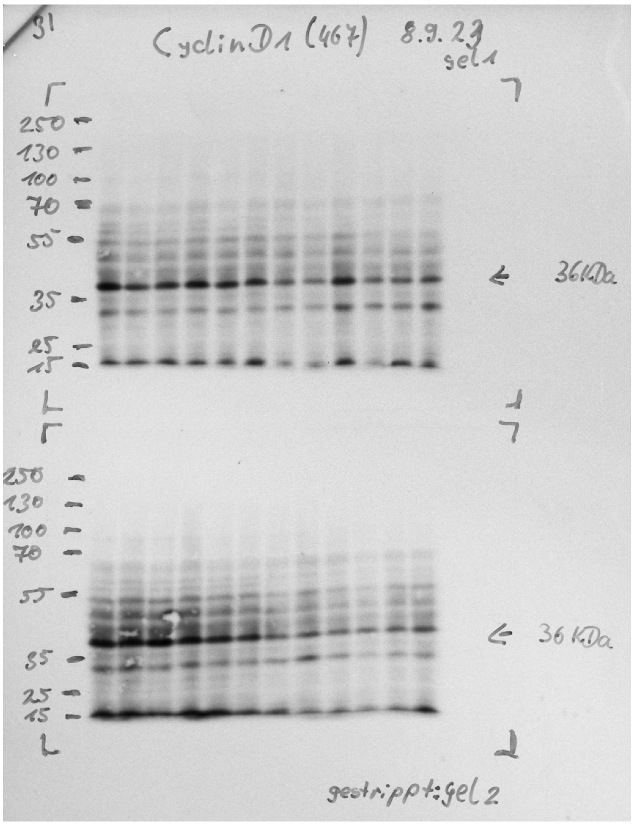

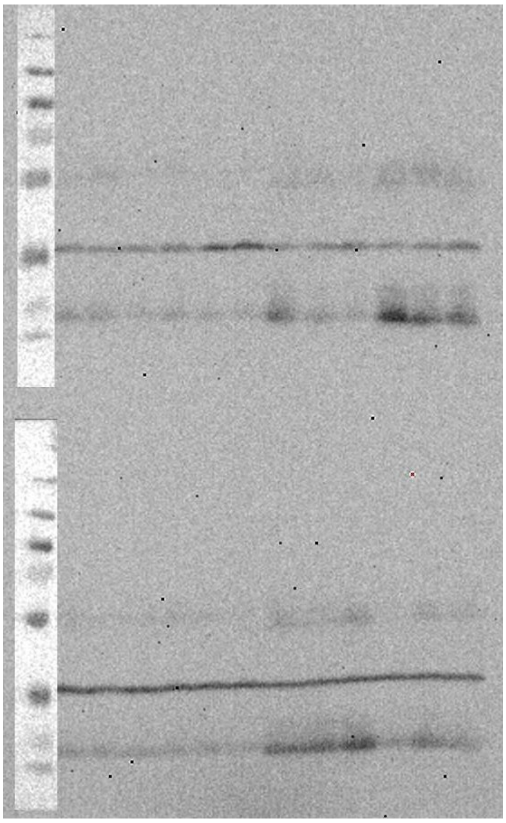

Original Blots

Suppl. Fig 8A Catalase (Intas ECL Chemostar)

GAPDH (Intas ECL Chemostar)

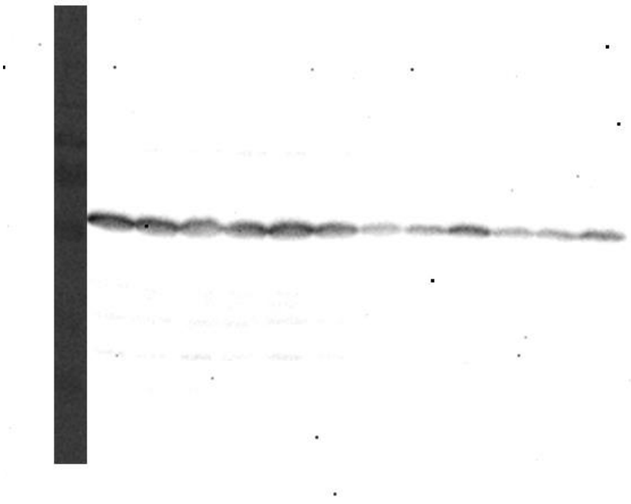

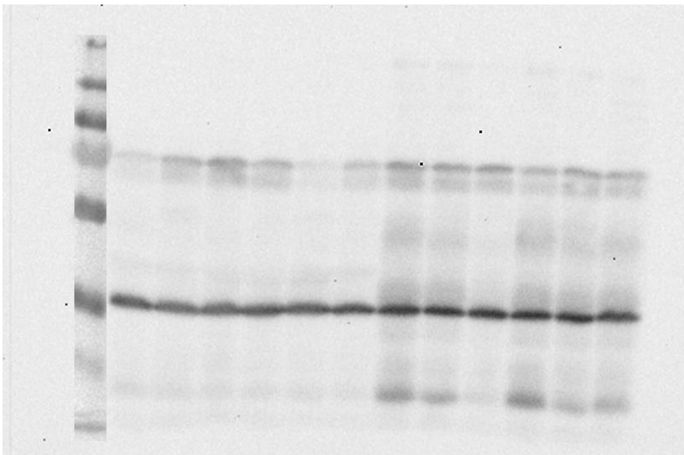

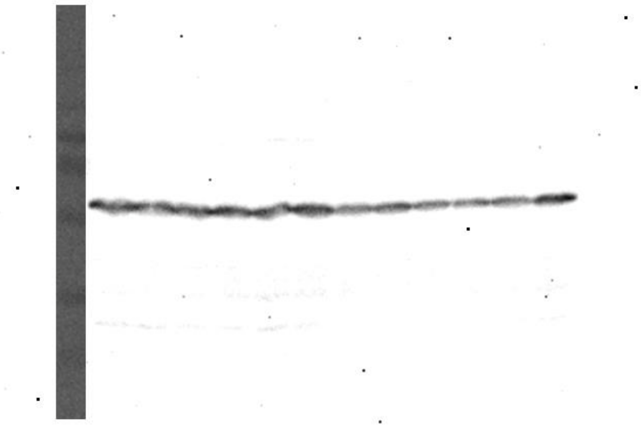

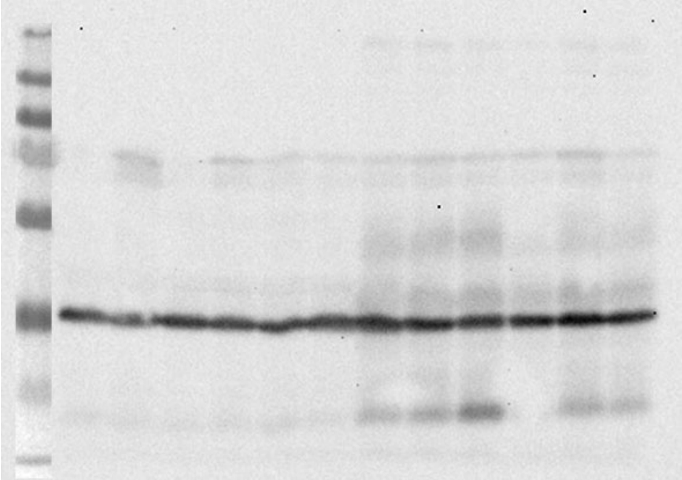

Repetitions

Suppl. Fig 8A Catalase (Intas ECL Chemostar)

GAPDH (Intas ECL Chemostar)

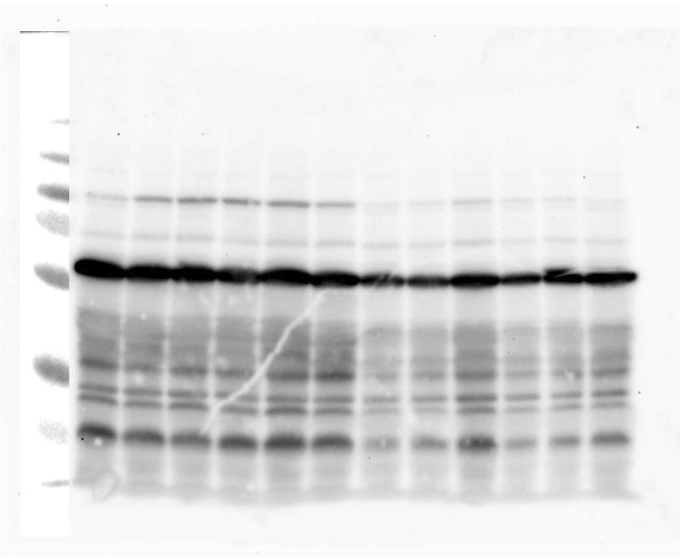

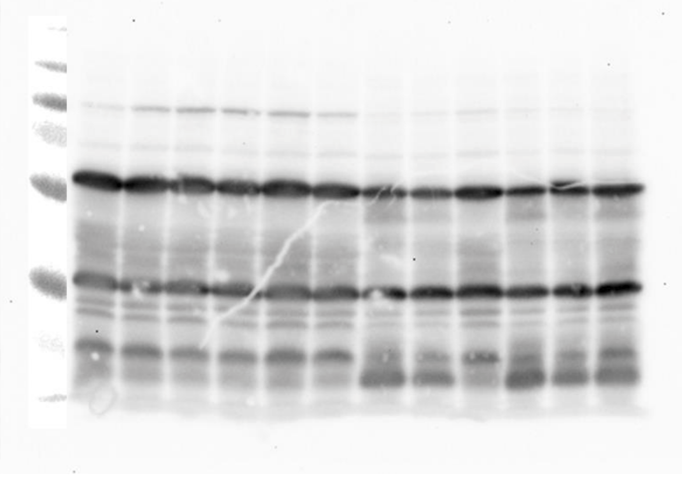

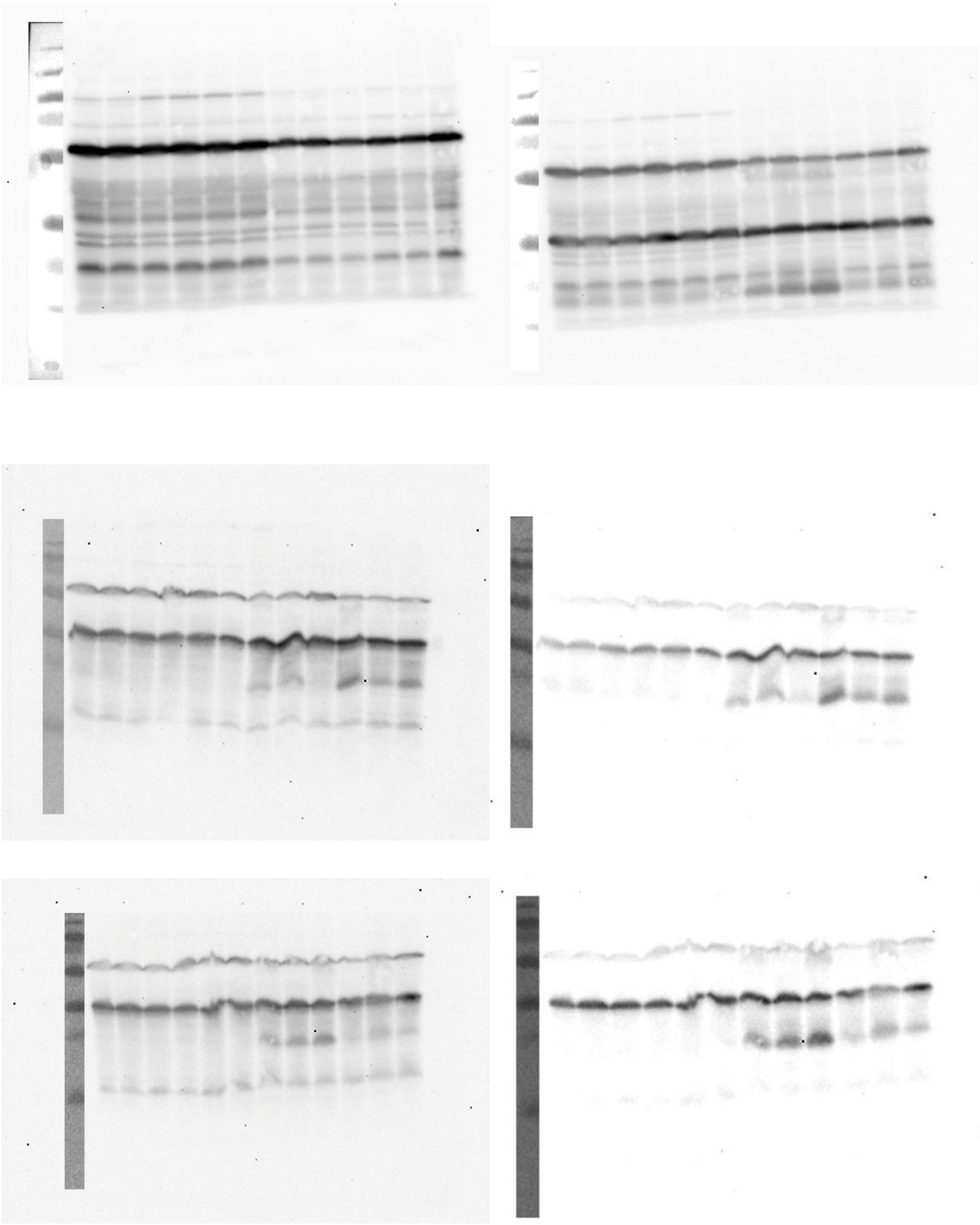

Original Blots

Suppl. Fig 8F BIP (Intas ECL Chemostar) GAPDH (Intas ECL Chemostar)

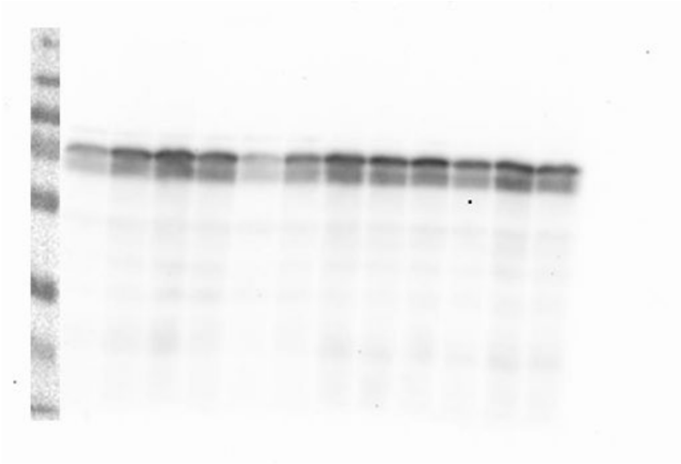
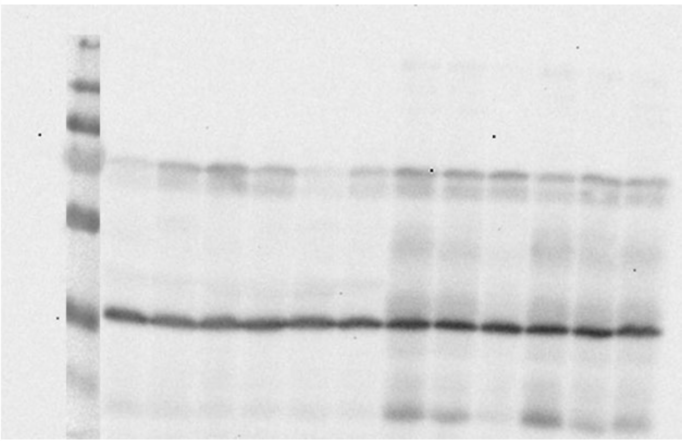

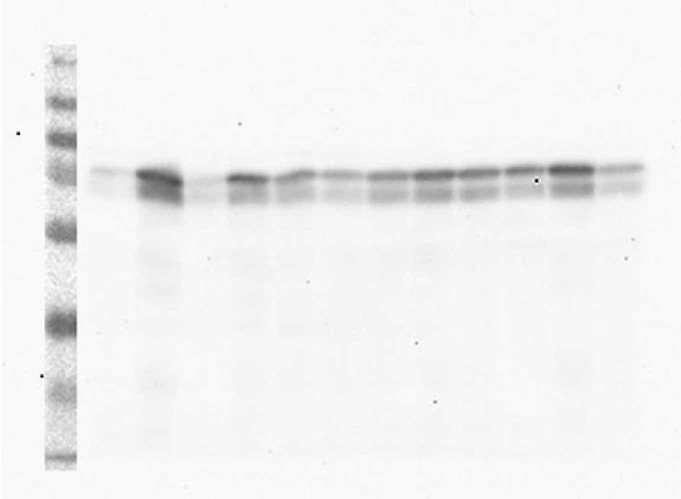
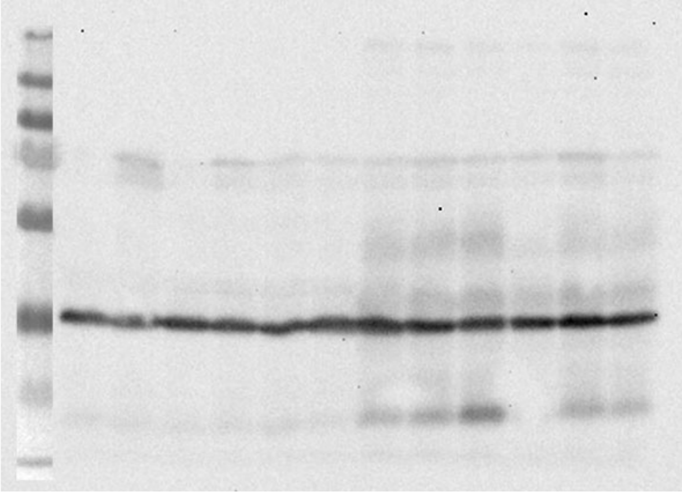

Repetitions

Suppl. Fig 8F BIP (Intas ECL Chemostar)

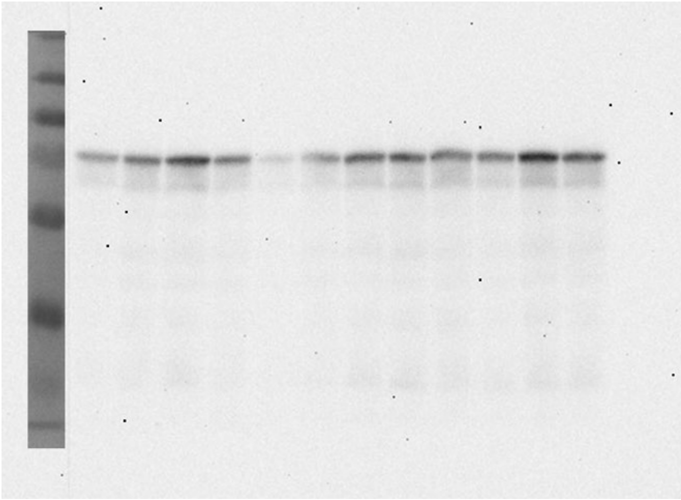

GAPDH (Intas ECL Chemostar)

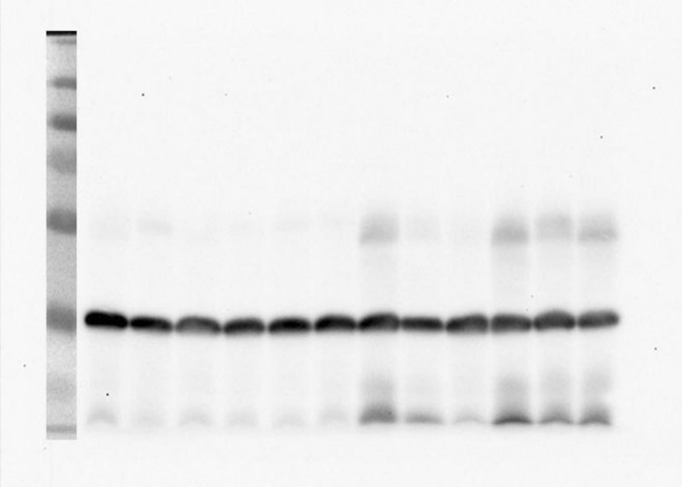

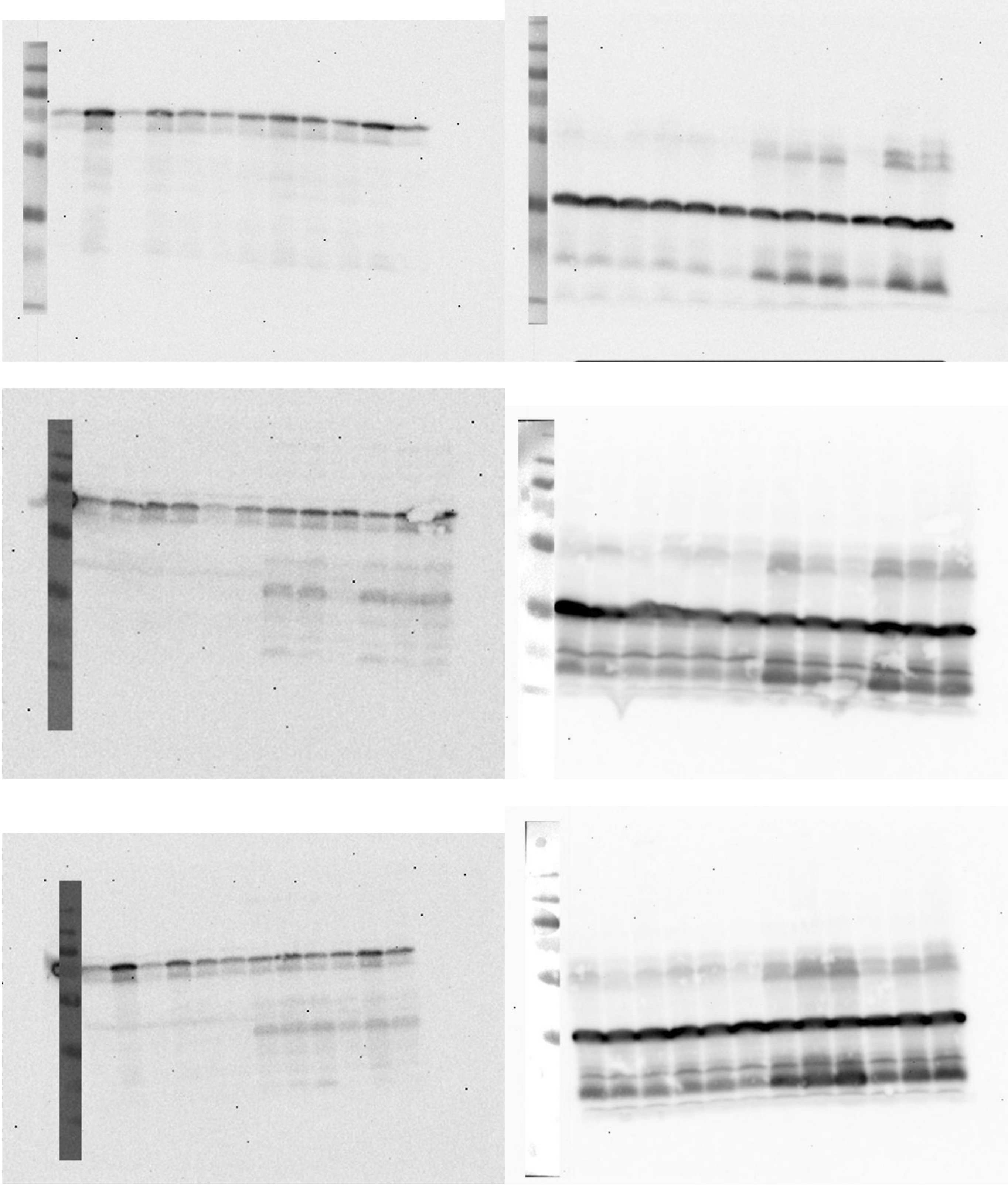

Original Blots

Suppl. Fig 8F eIF2a (Intas ECL Chemostar) GAPDH (Intas ECL Chemostar)

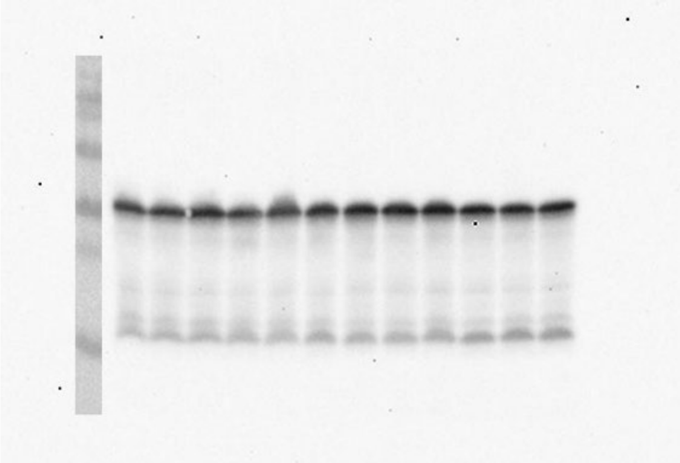

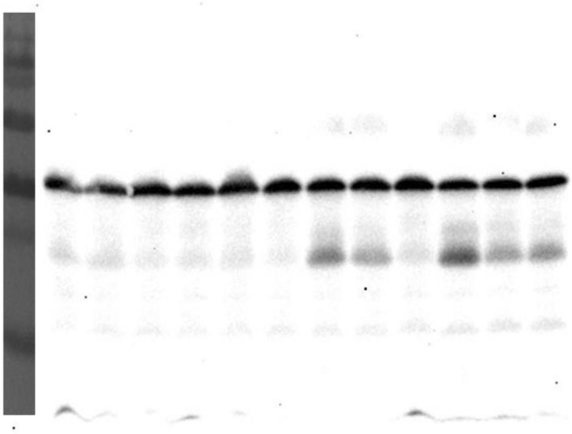

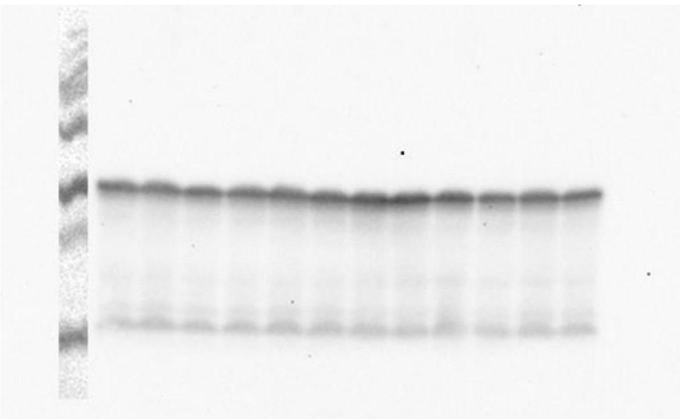

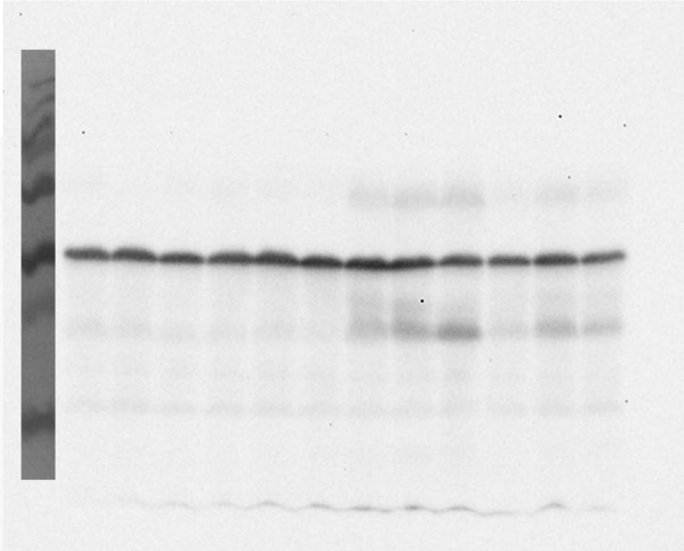

Repetitions

Suppl. Fig 8F eIF2a (Intas ECL Chemostar)

GAPDH (Intas ECL Chemostar)

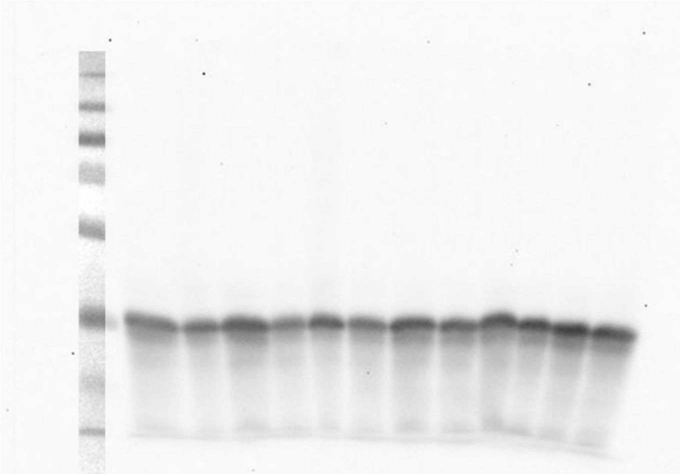

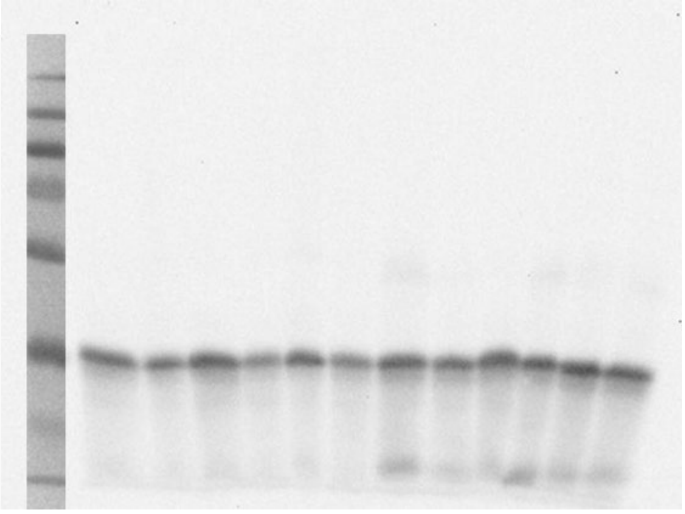

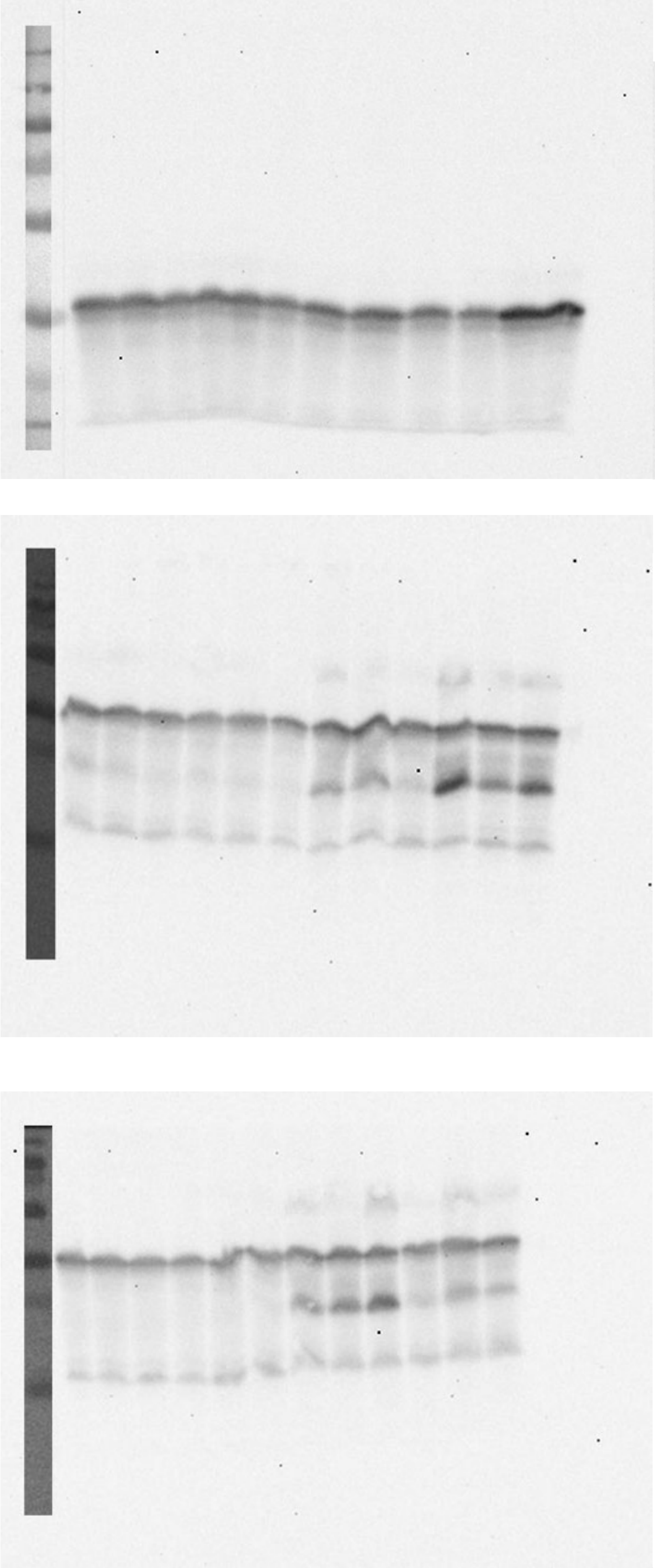

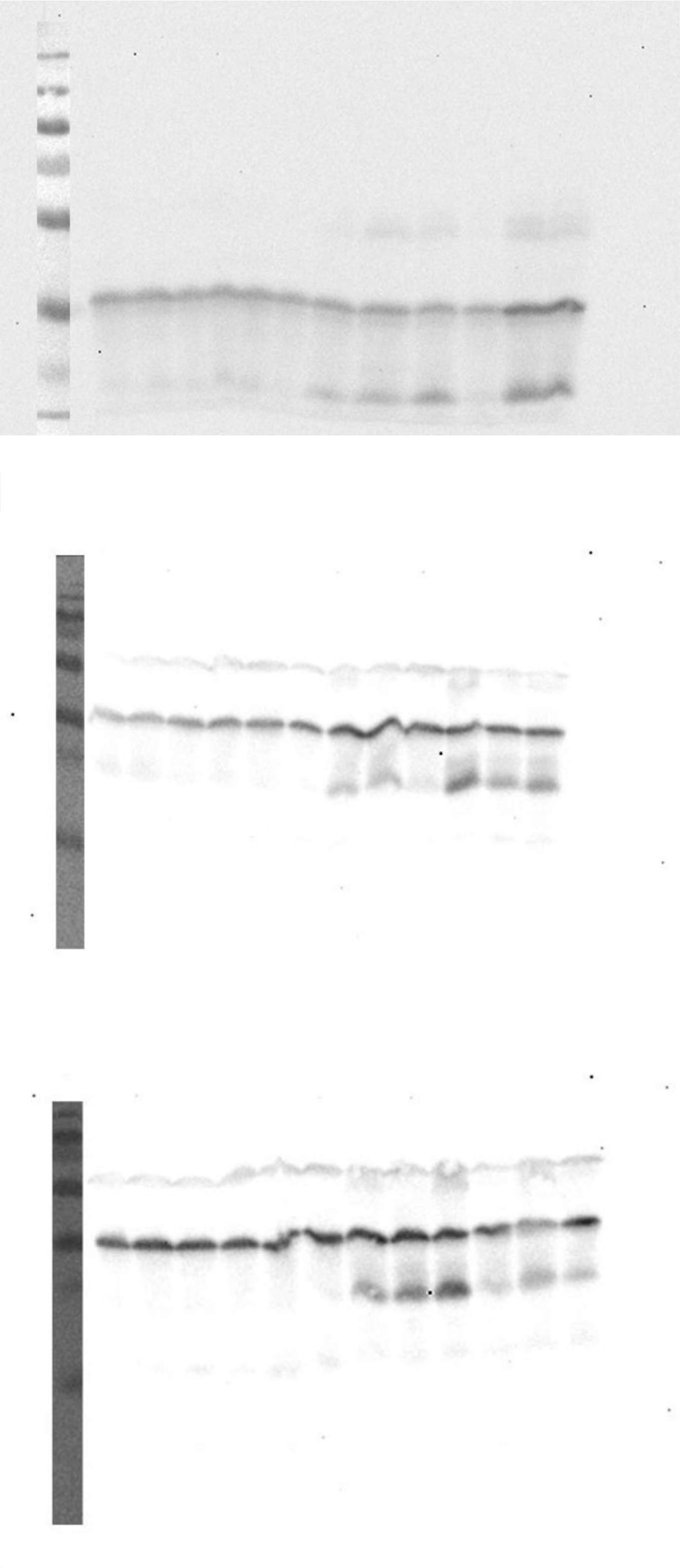

Original Blots

Suppl. Fig 8F p-eIF2a (Intas ECL Chemostar)

GAPDH (Intas ECL Chemostar)

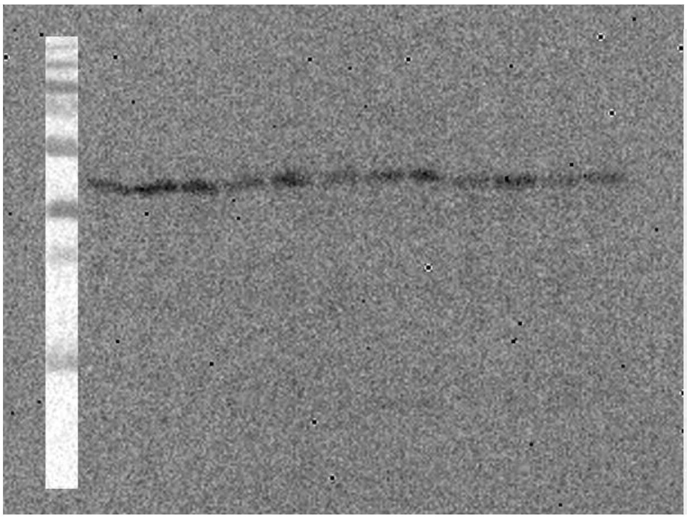

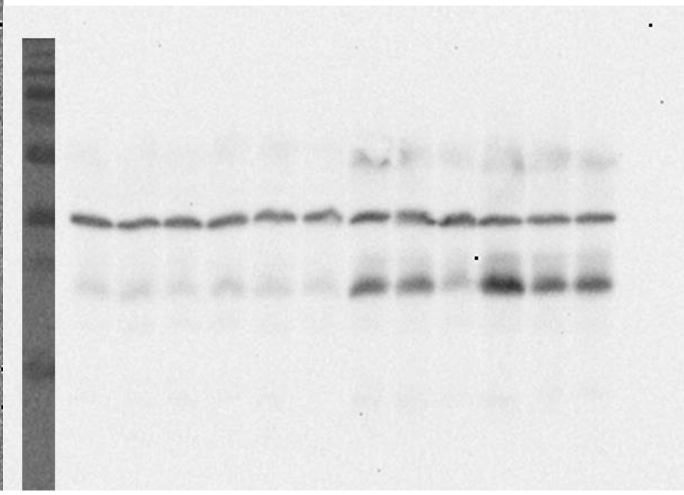

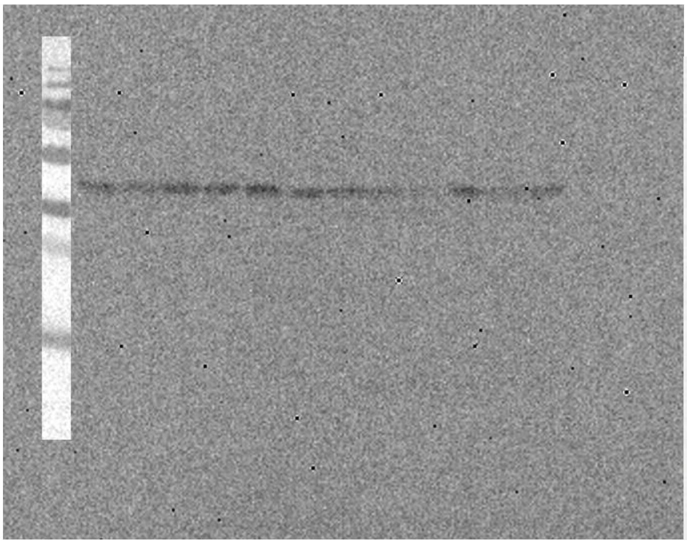

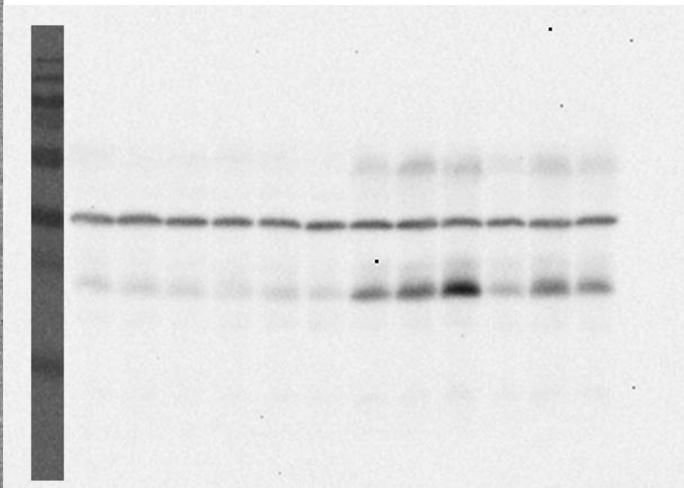

Repetitions

Suppl. Fig 8F p-eIF2a (Intas ECL Chemostar)

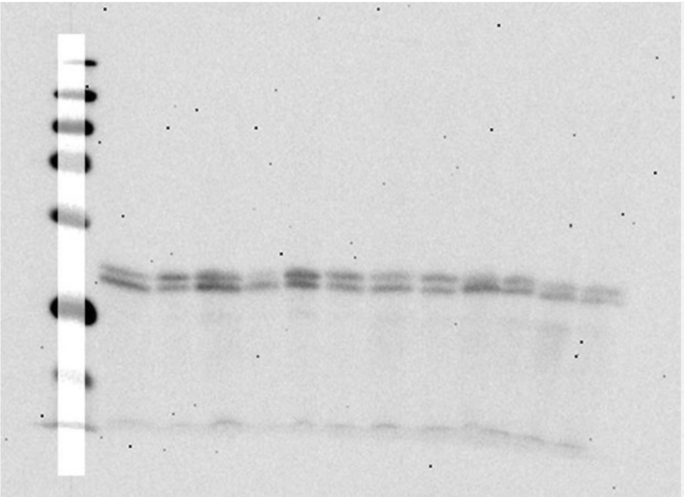

GAPDH (Intas ECL Chemostar)

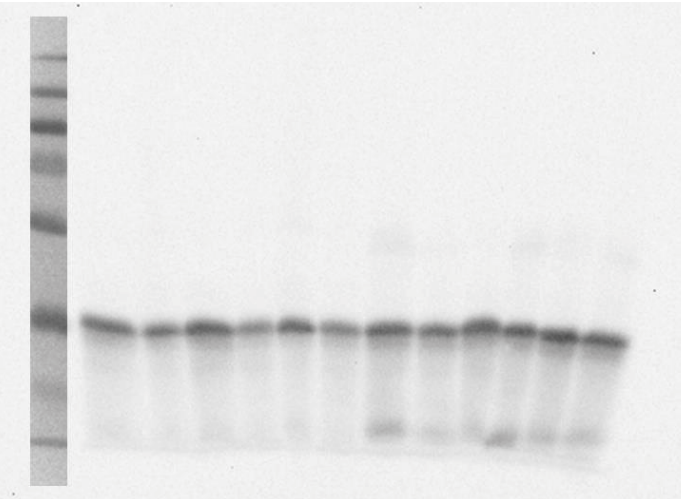

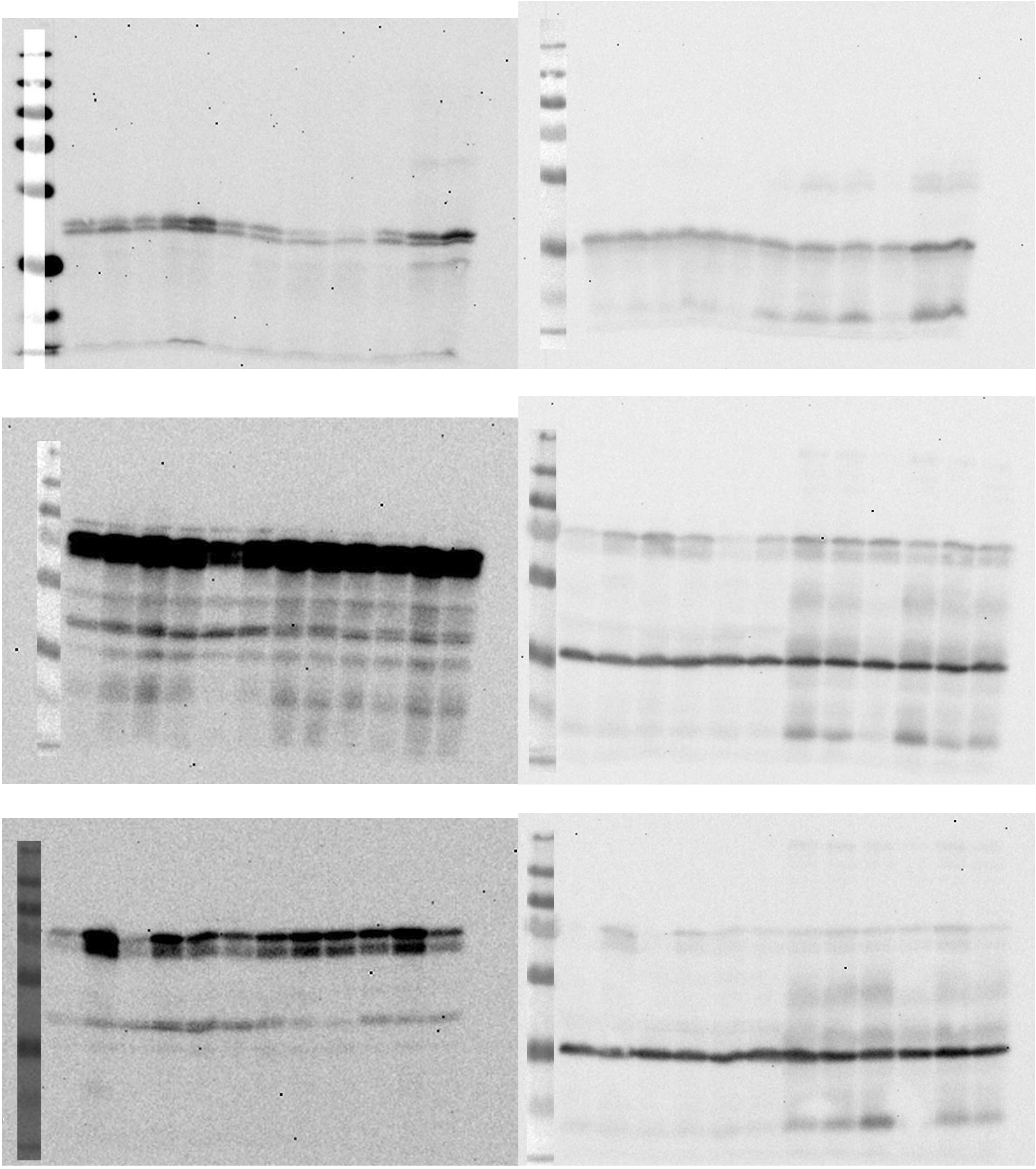

Original Blots

Suppl. Fig 12 PCNA (Intas ECL Chemostar) GAPDH (Intas ECL Chemostar)

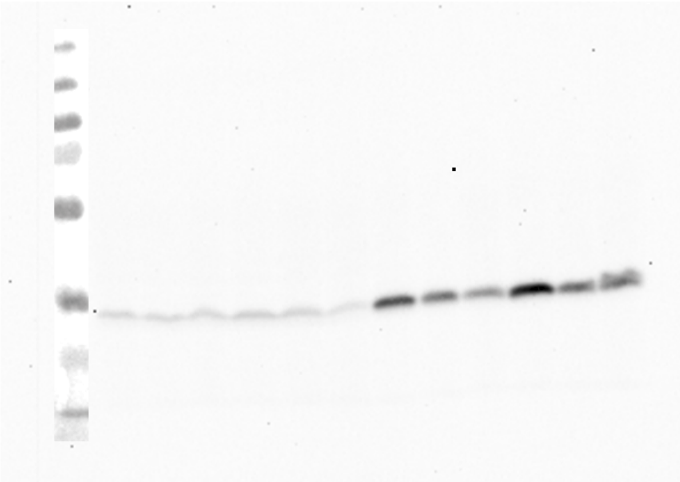

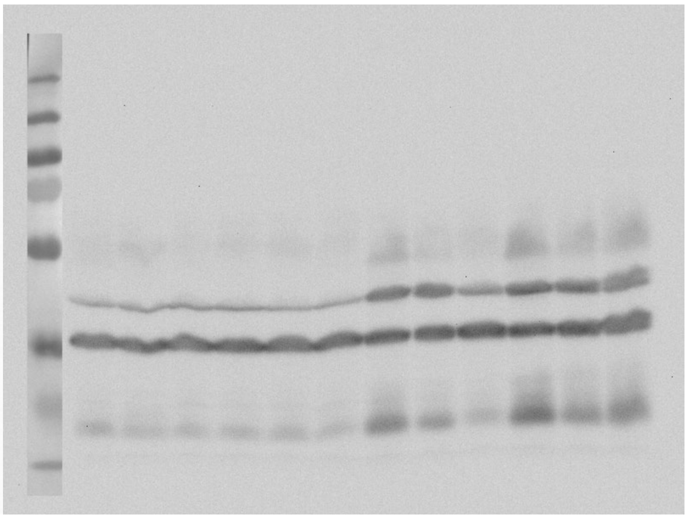

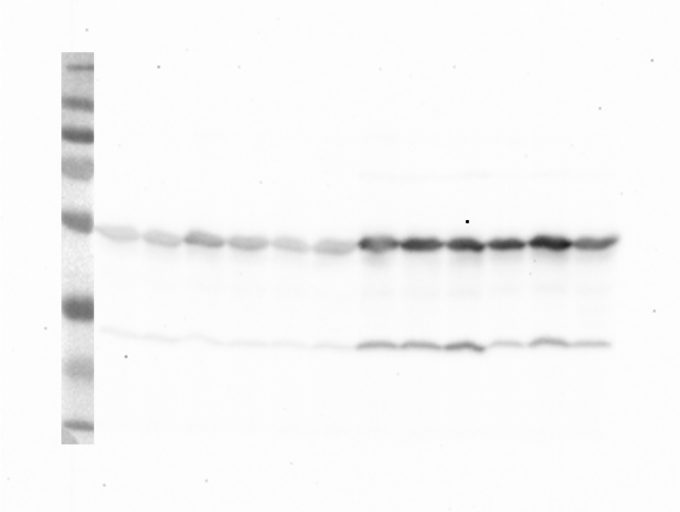

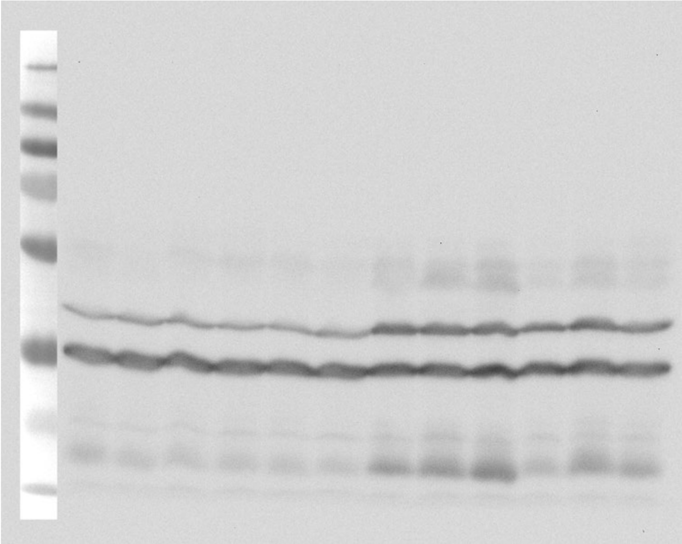

Repetitions

Suppl. Fig 12 PCNA (Intas ECL Chemostar)

GAPDH (Intas ECL Chemostar)

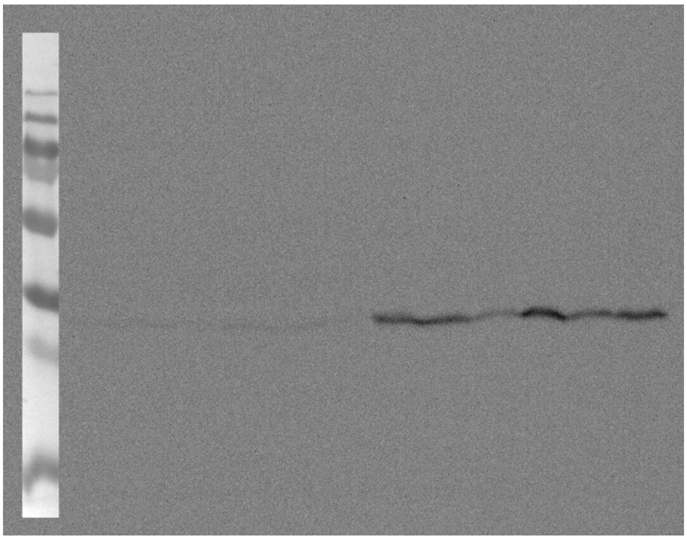

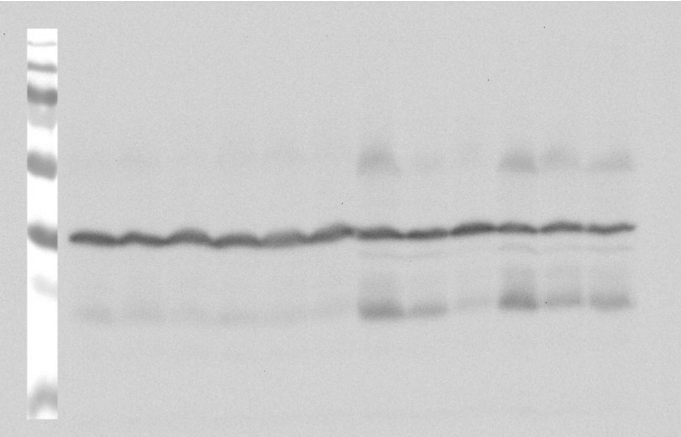

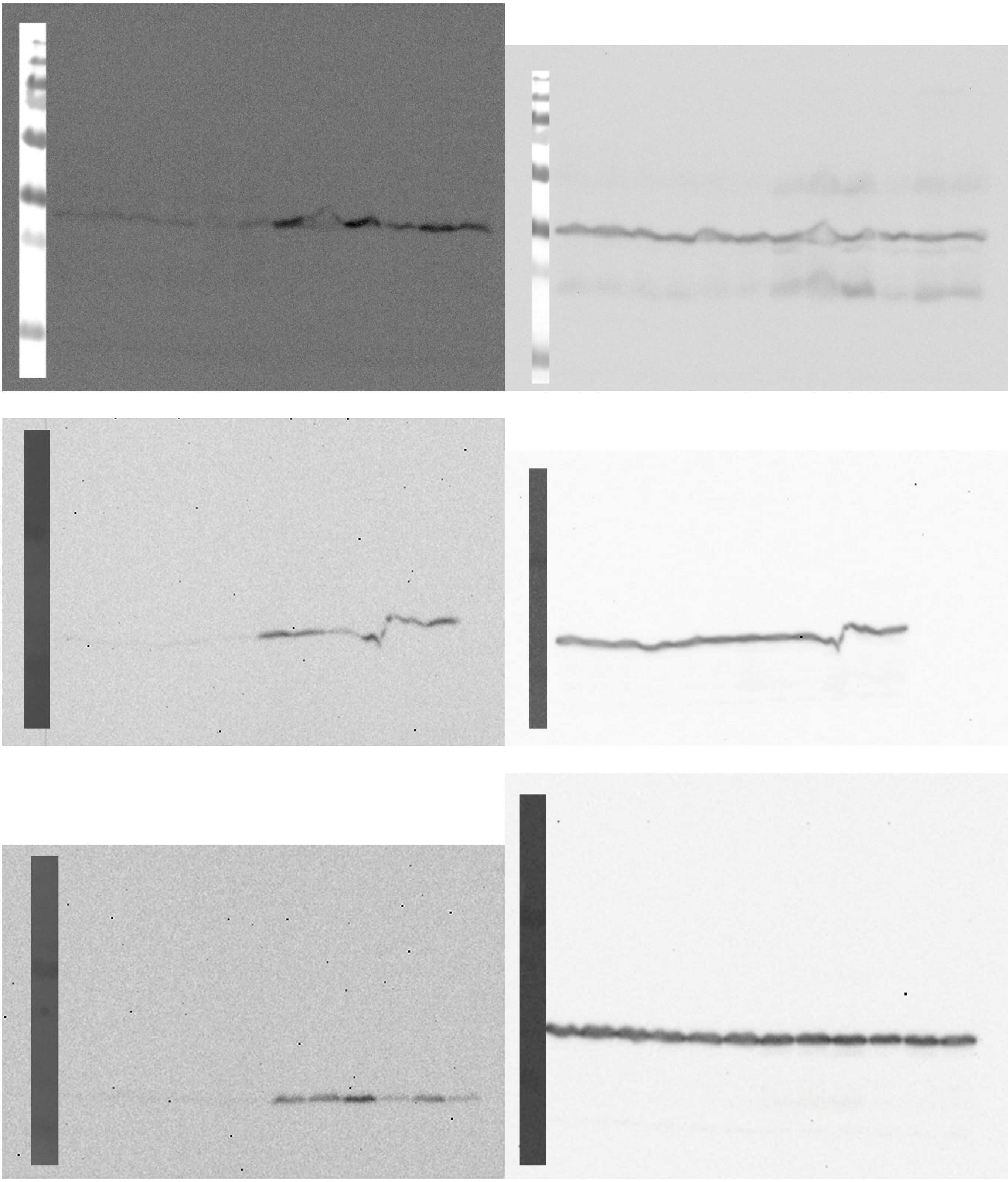

Supplement: Supplementary file 1 — Supplementary Figures. [file 41598_2023_47646_MOESM1_ESM.pdf]
